# Reliability assessment of markerless technologies in biomechanical motion analysis: a performance comparison

**DOI:** 10.3389/fspor.2025.1712332

**Published:** 2026-01-12

**Authors:** Ibrahim Cem Balci, Irem Sayin, Serkan Salturk, Rana Gursoy, Umut Ozsoy, Husnu Caglar Dogru, Gokhan Akca, Ali Eraslan, Onurcan Sahin, Ali Anil Demircali, Huseyin Uvet

**Affiliations:** 1 Department of Mechatronics Engineering, Yildiz Technical University, Istanbul, Türkiye; 2 Department of Informatics, Yildiz Technical University, Istanbul, Türkiye; 3 Department of Anatomy, Akdeniz University, Antalya, Türkiye; 4 Faculty of Communication/Radio, Television and Cinema, Canakkale Onsekiz Mart University, Canakkale, Türkiye; 5 Department of Sports Medicine, University of Health Sciences, Antalya Training and Research Hospital, Antalya, Türkiye; 6 Department of Metabolism, Digestion, and Reproduction, Faculty of Medicine, Imperial College London, London United Kingdom; 7 Istinye University, Artificial Intelligence Research and Application Center (YZAUM), Istanbul, Türkiye

**Keywords:** artificial intelligence in sports, body tracking, marker-based motion capture, markerless motion capture, motion analysis, pose estimation, reliability assessment, sports technology

## Abstract

**Introduction:**

Body tracking systems are utilized in various areas such as health and sports. In these areas, it is crucial to identify the most accurate and appropriate system for the intended design of the study.

**Methods:**

This study evaluates the reliability of markerless motion capture systems compared to a marker-based system (OptiTrack) as the gold standard. The systems assessed include StereoLabs ZED 2i, MediaPipe (2D and 3D), and YOLOv8 Pose (8n and 8x-p6). Simple upper and lower limb movements were analyzed under controlled conditions in a single participant. Intraclass Correlation Coefficient (ICC) values and Bland-Altman analysis were mainly used to evaluate agreement between systems, with some other techniques explored to provide additional perspective.

**Results:**

The results indicate that StereoLabs ZED 2i achieved the highest reliability among markerless systems (ICC: 0.92 for multi camera, 0.88 for single stereo camera), while MediaPipe 2D showed competitive performance (ICC: 0.85).

**Discussion:**

In providing comprehensive evaluations and uncovering performance differences that have been largely overlooked in the literature, our work not only questions existing paradigms but also lays a foundation for future research aimed at developing precise and scalable motion-tracking technologies. These findings contribute to understanding the applicability of markerless technologies in biomechanics.

## 1 Introduction

In recent years, advancements in motion capture technology have significantly transformed biomechanics, sports science, rehabilitation and even daily routine activities, such as prayer movements [1–3]. Marker-based and markerless motion capture technologies allow academics and practitioners to analyze and understand human movements. Each of these systems has unique advantages and limitations that affect its suitability for different applications.

Marker-based motion capture systems are considered the most reliable method for analyzing motion due to their exceptional accuracy and precision in capturing intricate motions [4, 5]. These systems employ numerous infrared cameras to identify passive reflective markers positioned on specified anatomical features of the participant’s body. The 3D coordinate data obtained from these systems offer extremely exact assessments of joint movements, making them suitable for applications that need precise motion analysis, such as gait analysis [6], and sports performance evaluation [7]. However, marker-based systems have certain disadvantages. Placing markers on individuals is time-consuming and requires skilled operators for precise placement and data collection. These systems are also relatively more expensive and require specialized space. They can suffer from problems such as occlusions and marker dropout, which can affect the accuracy of the data [8]. In addition, marker-based systems are restricted to indoor use because they rely on controlled environments. This requirement for a highly regulated setup limits their practical application.

However, recent advances in computer vision and deep learning have enabled markerless motion capture systems that estimate human pose directly from video without the need for physical markers. These systems typically combine commodity RGB or RGB-D cameras with 2D or 3D pose estimation networks, such as the MediaPipe framework [9] or more recent transformer-based architectures [10]. Comprehensive reviews have classified markerless pipelines according to hardware configuration (monocular/multi-view, RGB/depth), model architecture, and real-time performance, and have highlighted important trade-offs between accuracy, speed, and robustness in different environments [11, 12]. Building on these developments, the present work considers a set of widely available, low-cost markerless pipelines that are representative of different hardware and algorithmic choices.

Depth cameras such as the StereoLabs provide a balanced solution between traditional marker-based systems and purely RGB-based markerless approaches. By combining stereo RGB and depth sensing, these cameras can recover 3D joint positions within a relatively small capture volume while relying on inexpensive, portable hardware [13]. Depth-based setups have been used in tele-rehabilitation and remote functional assessment, where ease of deployment is critical [14]. Nevertheless, limited field of view, occlusions, and sensitivity to lighting and reflective surfaces can still restrict their effectiveness in specific applications, underscoring the need for systematic accuracy evaluations [15].

Numerous validation studies have compared markerless and marker-based motion capture in specific tasks. For gait and walking, several groups have reported small to moderate biases for some lower-limb angles alongside larger discrepancies for others, indicating that agreement is strongly joint- and condition-dependent [16–18]. In running and other dynamic tasks, 2D and 3D markerless pipelines have shown promising results for certain joint angles but also increased variability and sensitivity to occlusion and clothing [12, 19]. For upper-extremity movements, especially the shoulder, depth-based systems such as Azure Kinect have demonstrated good reliability and agreement with marker-based measurements in range-of-motion assessments [20, 21]. Markerless approaches have also been applied to balance-related tasks, where relatively small differences in static conditions contrast with larger discrepancies during more challenging dynamic movements [16, 22]. Beyond direct comparisons, recent work has focused on improving markerless accuracy via subject-specific modelling, trajectory smoothing, and anatomical landmark–driven keypoint estimation, as well as on developing more appropriate statistical frameworks for agreement analysis [4, 23, 24]. Collectively, these findings illustrate that markerless systems can approach marker-based performance under certain conditions, but their accuracy and robustness remain highly dependent on the specific task, camera configuration, and processing pipeline.

The decision between marker-based and markerless motion capture technologies depends on the accuracy requirements, context of use, and practical constraints of the application. Most existing validation studies have examined a single markerless system, often in multi-participant cohorts and primarily during gait or running, leaving open how different low-cost pipelines behave when tested under identical, highly controlled conditions. In particular, there is limited evidence on the performance of widely available depth-based and video-based systems for upper- as well as lower-extremity kinematics when inter-individual variability is minimised.

In the present work, we systematically compare a marker-based reference system (OptiTrack) with several representative markerless configurations: single- and multi-camera StereoLabs ZED 2i (hereafter referred to as StereoLabs) depth setups and single-camera pipelines based on MediaPipe and YOLOv8 Pose. Using an intensive single-subject, repeated-measures design, we analyze simple, predominantly single-degree-of-freedom (DoF) movements of the upper and lower extremities under standardised laboratory conditions. This design prioritises high-density data collection within a controlled framework to reduce confounding factors such as anthropometric variability and inconsistent movement execution, thereby isolating the instrument-level performance of each system relative to the gold standard. The central research question guiding this study is: to what extent do commonly used markerless motion capture systems provide reliable and accurate joint angle measurements compared to a marker-based gold standard? By answering this question, we provide a foundational comparison of instrument precision that can help clinicians and sports scientists select appropriate technologies based on baseline accuracy requirements, cost, and setup feasibility in their specific domains.

## 2 Materials and methods

The study employed an intensive repeated-measures, single-subject experimental design (SSED) focusing on the shoulder, elbow, hip, and knee joints. Data were collected from a single healthy female participant (age: 20 years, height: 165 cm, mass: 53 kg) with no known musculoskeletal or neurological impairments. The decision to utilize a single-subject design was methodological and deliberate for this foundational comparison. In biomechanical validation studies, inter-participant variability arising from differences in anthropometry, body composition, and individualized movement strategies often introduces significant confounding factors that can obscure the direct comparison of measurement technologies. By utilizing a single participant performing repeated trials, we ensure that the anatomical ground truth and the execution of the movement remain consistent across all tested systems. This approach maximizes internal validity, allowing for the isolation of measurement variance attributable solely to the motion capture technology itself, providing a high-fidelity assessment of instrument precision rather than population-level generalizability.

The participant provided written informed consent. This study was approved by the Ethics Committee for Clinical Research of the Faculty of Medicine, Akdeniz University, with decision number KAEK-200. The movements analyzed in this study were carefully selected by specialists to ensure suitability for controlled and repeatable analysis, focusing on elementary, single-DoF movements. Establishing the reliability of markerless systems during these standardized, isolated motions is a necessary prerequisite before assessing their performance in complex, multi-DoF, or high-velocity tasks. For the upper extremity, abduction/adduction, horizontal abduction/adduction, rotation movements were performed at the shoulder; flexion/extension movements were performed at the elbow. For the lower extremity, flexion/extension, abduction/adduction, rotation movements were performed at the hip; flexion/extension movements were performed at the knee. Motion capture operations were recorded synchronously with the OptiTrack marker camera set and the StereoLabs cameras with multi camera and single stereo cameras setups belonging to the StereoLabs company. First the performance of the StereoLabs body tracking model was examined according to the OptiTrack system, which is accepted as the gold standard due to their high level of precision [15]. Then, the performance of MediaPipe and YOLOv8 Pose was examined according to the gold standard system. Two different models of the YOLOv8 Pose architecture were used. The YOLOv8n-pose [8n] model stands out with its high processing speed and low accuracy rate. This model is especially preferred in real-time applications. The other model is the YOLOv8x-pose-p6 [8x-p6] model; although this model works slower, it offers higher accuracy and is used in scenarios that require detailed analysis and precision.

### 2.1 Motion capture systems and calibration process

OptiTrack is a 3D tracking system frequently used in industrial and scientific fields. The system offers both marker-based and markerless tracking solutions. In this study, a marker-based system was chosen to achieve more precise and reliable motion tracking. OptiTrack stands out for its superior accuracy and reliability, especially in scenarios that require detailed and high-precision motion tracking. OptiTrack is used in various fields, such as animation, sports analysis, medical research, robotics, and VR/AR applications. This study utilized a system consisting of 16 cameras with a frame rate of 240 frames per second and 49 passive markers of 14 mm in diameter.

**Figure 1:**
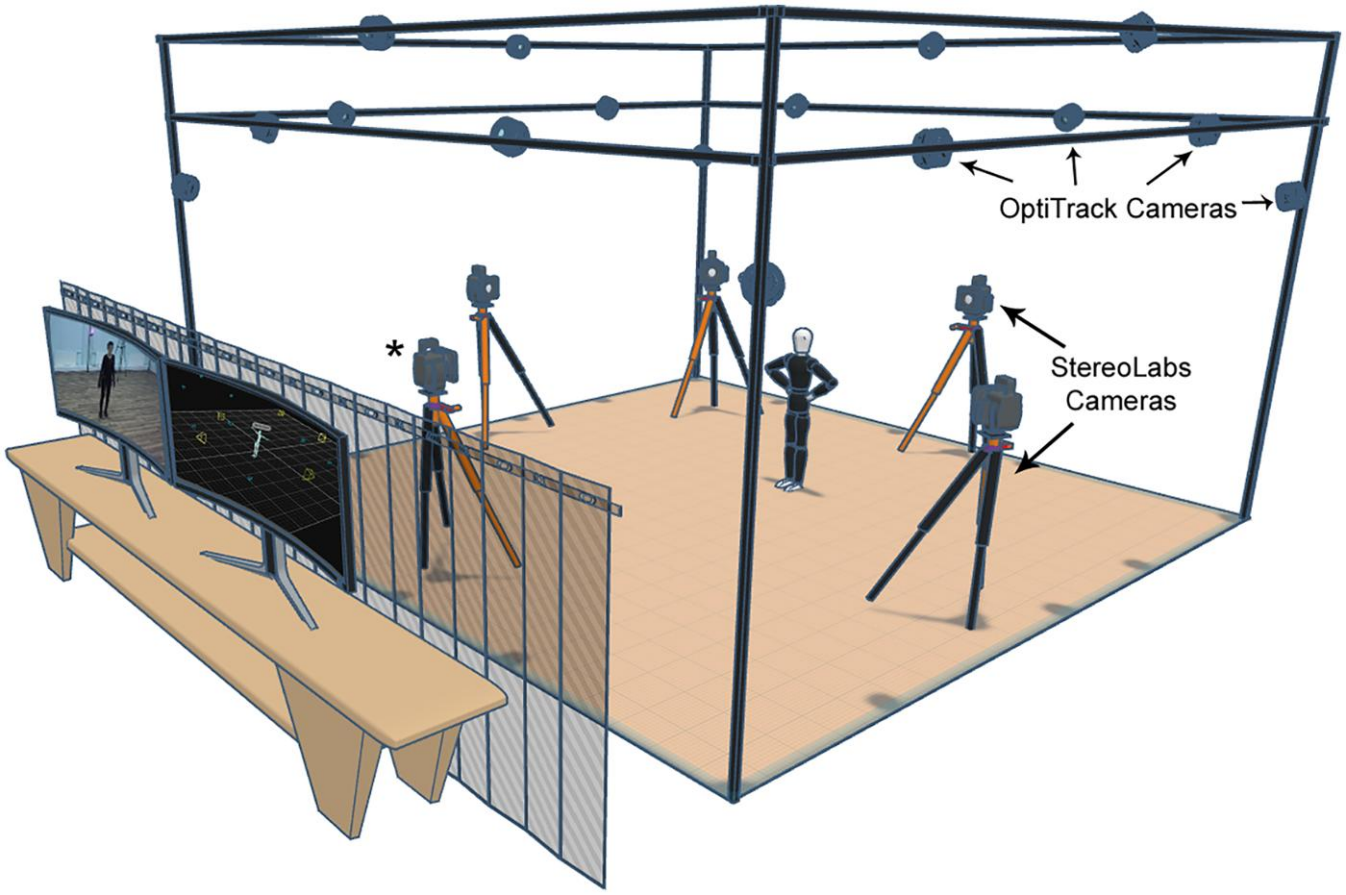
Layout of OptiTrack and StereoLabs cameras.

The necessary static calibration procedures for installing the OptiTrack system have been completed, and the cameras have been positioned. The static calibration involves placing a marker on the ground to help position the cameras correctly. After that, the T-bar was moved within the view of all the cameras. This process continued until each camera collected enough data, allowing the system to calculate the relative positions of the cameras. This step completed the dynamic calibration. Finally, to determine the central point, a calibration square indicating the x, y, and z planes was placed on the ground, defining the ground plane and the global origin. This origin was marked with black tape. Following this, the participant’s movements were tracked based on this global origin point. The environment in which the study was conducted is shown in Figure 1. Five synchronized StereoLabs cameras were chosen to compare markerless systems. The synchronization between cameras is done with the tools provided by the ZED SDK [13]. StereoLabs cameras offer flexible imaging options with different resolutions and frame rate settings. In this study, a 720p resolution and 60 FPS setting were preferred. This setting allowed for smooth motion tracking while balancing the processing load.

For the calibration of the StereoLabs system, the ZED360 software provided by StereoLabs was applied after the cameras were positioned. To calibrate the system, once all cameras were in place, a person walked within the camera boundaries to ensure the system’s recorded camera positions matched their actual locations. Additionally, for calibration to be fully completed, the StereoLabs’ internal optimization algorithm minimizes the spatial discrepancy between the skeletons detected by individual cameras and the fused global skeleton, ensuring that the spatial alignment and superimposition of anatomical landmarks across multiple views reach the software’s quality thresholds. This process can take 1 to 5 min, and a JSON file containing the cameras’ positions and orientations is generated at the end. The camera placements demonstrated in the study are shown in Figure 1, including the positions of the OptiTrack cameras and the StereoLabs cameras. Camera with the “*” symbol, as indicated in Figure 1, was used for processing single-camera models.

### 2.2 Placement of marker set

**Figure 2:**
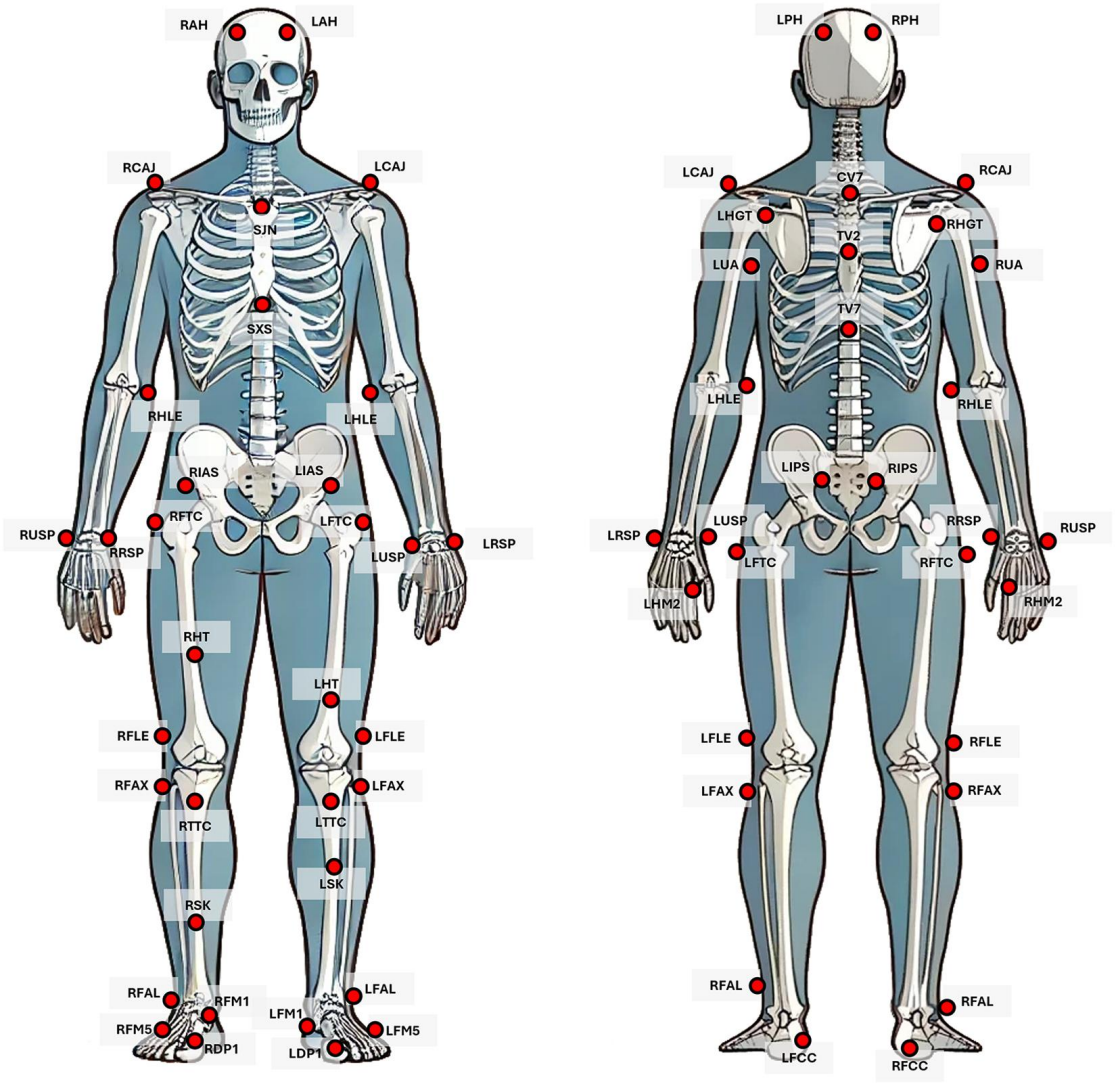
Location of markers at anterior and posterior views [27].

Forty-nine passive 14 mm markers were utilized for the marker-based motion capture system. The placement of 49 markers was done according to the documentation of OptiTrack [25–27]. The region and placement of the markers used are as follows, as shown in Figure 2: (1) Head: left and right anterior head (LAH/RAH), left and right posterior head (LPH/RPH); (2) Torso: sternum jugular notch (SJN), sternum xiphoid process (SXS), cervical spine vertebra 7 (CV7), thoracic spine vertebra 2 and 7 (TV2-TV7), left and right glenohumeral joint (LHGT/RHGT); (3) Waist: left and right iliac anterior spine (LIAS/RIAS), left and right iliac posterior spine (LIPS/RIPS); (4) Upper Extremity: left and right clavicle-acromion joint (LCAJ/RCAJ), left and right humerus lateral epicondyle (LHLE/RHLE), left and right upper arm (LUA/RUA); (5) Hand: left and right hand second metacarpal (LHM2/RHM2), left and right ulna styloid process (LUSP/RUSP), left and right radius styloid process (LRSP/RRSP); (6) Lower Extremity: left and right femoral greater trochanter (LFTC/RFTC), left and right femur lateral epicondyle (LFLE/RFLE), left and right thigh (LTH/RTH), left and right superior knee (LSK/RSK), left and right tibial tubercle (LTTC/RTTC), left and right fibula apex (LFAX/RFAX); (7) Foot: left and right fibula ankle lateral (LFAL/RFAL), left and right foot fifth metatarsal (LFM5/RFM5), left and right foot first metatarsal (LFM1/RFM1), left and right foot calcaneus (LFCC/RFCC), left and right first distal phalanx (LDP1/RDP1).

### 2.3 Processing and analyzing data

The data for the marker-based system was collected using the OptiTrack Motive application. For comparisons with markerless systems, videos were recorded using five StereoLabs cameras. These recorded videos were later processed, and information about body tracking results from StereoLabs was collected and saved. Data from both multiple cameras and a single stereo camera were used to examine the StereoLabs system. Similarly, for MediaPipe and YOLOv8 Pose, the same video recordings from the StereoLabs camera system were processed, but only one of the camera recordings was used this time. Data from StereoLabs, MediaPipe, and YOLOv8 Pose were matched according to the frame counts of the videos. This data from markerless methods was then aligned with the data from OptiTrack based on the data’s timestamps. Since data from both systems were collected at different frame rates (OptiTrack at 240 FPS and StereoLabs at 60 FPS), temporal synchronization was critical. While hardware synchronization was not feasible given the disparate systems, synchronization was achieved computationally post-acquisition. We implemented a nearest-neighbor timestamp matching algorithm, aligning each StereoLabs frame with the most temporally corresponding OptiTrack frame. Given the high sampling frequency of the OptiTrack system, the maximum potential temporal misalignment was limited to approximately ±2.08 ms (half the interval of a 240 Hz signal). This level of temporal discrepancy is considered negligible for the slow, controlled movements analyzed in this study.

During the calculation of body angles, the angles of the vectors formed by the joints relative to each other were examined. While collecting data, the participant was asked to perform a single movement at a time, allowing movements to have distinct starting and ending times, making separating the movements easier. Each tested movement was repeated five times, and the data from these repetitions were used to compute reliability metrics within each system. This repetition allowed us to perform statistical agreement analysis despite the single-participant design. The participant was asked to repeat each movement five times, with distinct starting and ending points including shoulder abduction/adduction, horizontal abduction/adduction, and rotation; elbow flexion/extension; hip flexion/extension, abduction/adduction, and rotation; and knee flexion/extension. All movements were performed under the guidance of experts and were selected based on their recommendations. The participant was instructed to ensure that only one joint was actively moving during each movement to isolate joint-specific angles. For shoulder abduction/adduction, the angle between the vector formed by the elbow and shoulder and the vector formed by the shoulder and hip on the relevant side was examined. For the elbow angle, the angle between the vector formed by the shoulder and elbow and the vector formed by the elbow and wrist was examined. Angles for other regions were calculated similarly, and all systems were compared in this regard.

### 2.4 Statistical analysis

Intraclass correlation coefficient (ICC) values were examined for statistical analysis. In the comparison, the gold standard system was accepted as the reference and the compatibility of the other systems was examined. The participant was asked to repeat each movement five times and the angles calculated throughout the process were compared. The 95% confidence interval (CI) of the ICC estimate categorizes values below 0.5 as poor reliability, values between 0.5 and 0.75 as moderate reliability, values between 0.75 and 0.9 as high reliability, and values over 0.90 as outstanding reliability [28]. The reliability of the approaches was assessed using the ICC (2, k). However, it is crucial to note that within this single-subject repeated-measures design, these metrics quantify the technical consistency of the markerless systems relative to the gold standard for a specific morphology. Consequently, the reported values reflect intra-subject reliability and may be prone to subject-specific biases (e.g., anthropometry, movement execution); therefore, they should not be interpreted as evidence of population-level validity. In addition, for each markerless system we report an average ICC value, defined as the arithmetic mean of the ICCs across all joint movement combinations analyzed for that system. These averaged ICCs are used as descriptive summary indicators and are always interpreted together with the corresponding bias and limits of agreement. Furthermore, Bland-Altman analysis were employed to assess the agreement between the methods. Statistical analysis were performed using the Pingouin package [29] in Python.

Limits of Agreement (LoA), ICC, SEM (Standard Error of Measurement) and MDC (Minimal Detectable Change) were calculated as comparison metrics. Bias, LoA, SEM, MDC metrics have the same unit as the tested measurement and for this study this is an angle in degrees. The equation for calculating bias is given in Equation 1.

$$\text{Bias}=\frac{1}{n}\sum_{i=1}^{n}\left(y_{1i}-y_{0i}\right)$$ (1)

where n is the number of data points, $y_{1}$ is the compared method, and $y_{0}$ is the gold standard. The equation for calculating LoA is given in Equation 2.

LoA=Bias±1.96×SD (2)

where SD indicates the standard deviation. The overall ratio of data within the LoA range is calculated using Equation 3.

$${\text{LoA}}_{\text{Ratio}}=\frac{N_{\text{inside}}}{N_{\text{total}}}$$ (3)

where $N_{\text{inside}}$ indicates the number of data points within the LoA range, and $N_{\text{total}}$ indicates the total number of data points. The Standard Error of Measurement (SEM) is calculated using the ICC as shown in Equation 4.

$$\text{SEM}=\mathrm{SD}\times \sqrt{1-\text{ICC}}$$ (4)

The equation for the MDC calculation [30] is provided in Equation 5.

$$\text{MDC}=1.96\times \sqrt{2}\times \text{SEM}$$ (5)

## 3 Results

To characterize the relative reliability of each markerless system across all joint–movement conditions, we report both the range of ICC values and a mean ICC summarising its overall performance. In the comparison made against the system accepted as the gold standard, the ICC values were calculated as between 0.71 and 0.98 (average: 0.92) for the system built with StereoLabs multi cameras; between 0.24 and 0.99 (average: 0.88) for the system built with StereoLabs single stereo camera; between 0.47 and 0.98 (average: 0.85) for the MediaPipe [2D] model; between 0.27 and 0.96 (average: 0.66) for the MediaPipe [3D] model; between -0.03 and 0.98 (average: 0.61) for the YOLO Pose [8n] model; and between 0.20 and 0.98 (average: 0.74) for the YOLO Pose [8x-p6] model. The values mentioned here are the lowest and highest ICC values obtained as a result of each motion and the average of all ICC values of motions obtained for the model.

In comparisons made for the shoulder; similar ICC values were obtained for right shoulder abduction/adduction (StereoLabs [Multi]: 0.98, StereoLabs [Single]: 0.98, MediaPipe [2D]: 0.98, MediaPipe [3D]: 0.96, YOLO Pose [8n]: 0.98, YOLO Pose [8x-p6]: 0.98) and left shoulder abduction/adduction (StereoLabs [Multi]: 0.97, StereoLabs [Single]: 0.96, MediaPipe [2D]: 0.95, MediaPipe [3D]: 0.93, YOLO Pose [8n]: 0.95, YOLO Pose [8x-p6]: 0.95). StereoLabs had higher ICC values for right shoulder horizontal abduction/adduction (StereoLabs [Multi]: 0.94, StereoLabs [Single]: 0.88, MediaPipe [2D]: 0.77, MediaPipe [3D]: 0.32, YOLO Pose [8n]: 0.82, YOLO Pose [8x-p6]: 0.79), right shoulder rotation (StereoLabs [Multi]: 0.98, StereoLabs [Single]: 0.91, MediaPipe [2D]:0.94 , MediaPipe [3D]: 0.89, YOLO Pose [8n]: 0.61, YOLO Pose [8x-p6]: 0.42), left shoulder horizontal abduction/adduction (StereoLabs [Multi]: 0.96, StereoLabs [Single]: 0.97, MediaPipe [2D]: 0.88, MediaPipe [3D]: 0.82, YOLO Pose [8n]: 0.86, YOLO Pose [8x-p6]: 0.85), left shoulder rotation (StereoLabs [Multi]: 0.98, StereoLabs [Single]: 0.96, MediaPipe [2D]: 0.96, MediaPipe [3D]: 0.82, YOLO Pose [8n]: 0.84, YOLO Pose [8x-p6]: 0.95) (Table 1). In addition, when MDC and SEM values are examined, StereoLabs has lower values for right shoulder horizontal abduction/adduction, right shoulder rotation, left shoulder horizontal abduction/adduction, left shoulder rotation (Statistical metrics other than Bias, ICC, and LoA (LoA Ratio, 95% CI, SEM, MDC) are available in the Supplementary Material.).

**Table 1:** Reliability between methods w.r.t. golden standard for shoulder.

Motion	Metric	StereoLabs [Multi]	StereoLabs [Single]	MediaPipe [2D]	MediaPipe [3D]	YOLO pose [8n]	YOLO pose [8x-p6]
RSAA	Bias	−2.03	6.55	3.82	7.27	5.22	3.87
	LoA	−32.23 to 28.17	−16.86 to 29.96	−24.24 to 31.88	−31.27 to 45.81	−22.97 to 33.42	−23.77 to 31.51
	ICC	0.98	0.98	0.98	0.96	0.98	0.98
RSHAA	Bias	−11.19	−13.18	−28.71	−1.15	−22.23	−21.18
	LoA	−40.15 to 17.77	−58.97 to 32.61	−91.89 to 34.47	−71.71 to 69.41	−76.90 to 32.45	−90.39 to 48.04
	ICC	0.94	0.88	0.77	0.32	0.82	0.79
RSR	Bias	−4.89	21.44	−0.81	−23.45	3.80	−3.79
	LoA	−37.13 to 27.34	−25.81 to 68.70	−68.33 to 66.72	−77.79 to 30.90	−96.42 to 104.02	−119.40 to 111.83
	ICC	0.98	0.91	0.94	0.89	0.61	0.42
LSAA	Bias	−1.35	1.69	0.67	9.92	3.61	2.24
	LoA	−36.72 to 34.03	−37.40 to 40.78	−44.71 to 46.04	−42.44 to 62.27	−40.02 to 47.24	−43.15 to 47.62
	ICC	0.97	0.96	0.95	0.93	0.95	0.95
LSHAA	**Bias**	−5.61	−1.08	4.95	6.63	4.00	−1.75
	LoA	−34.24 to 23.02	−27.28 to 25.11	−43.38 to 53.28	−39.95 to 53.21	−40.85 to 48.85	−62.21 to 58.71
	ICC	0.96	0.97	0.88	0.82	0.86	0.85
LSR	Bias	−5.63	−13.04	−0.57	−20.94	−25.09	−1.23
	LoA	−40.23 to 28.98	−49.17 to 23.10	−49.47 to 48.33	−84.94 to 43.07	−94.19 to 44.01	−54.75 to 52.28
	ICC	0.98	0.96	0.96	0.82	0.84	0.95

RSAA, Right Shoulder Abduction/Adduction; RSHAA, Right Shoulder Horizontal Abduction/Adduction; RSR, Right Shoulder Rotation; LSAA, Left Shoulder Abduction/Adduction; LSHAA, Left Shoulder Horizontal Abduction/Adduction; LSR, Left Shoulder Rotation.

In the comparisons made for the elbow, StereoLabs has higher ICC values for right elbow flexion/extension (StereoLabs [Multi]: 0.97, StereoLabs [Single]: 0.99, MediaPipe [2D]: 0.95, MediaPipe [3D]: 0.61, YOLO Pose [8n]: 0.89, YOLO Pose [8x-p6]: 0.95), left elbow flexion/extension (StereoLabs [Multi]: 0.96, StereoLabs [Single]: 0.86, MediaPipe [2D]: 0.94, MediaPipe [3D]: 0.42, YOLO Pose [8n]: 0.57, YOLO Pose [8x-p6]: 0.94) (Table 2). In addition, when the MDC and SEM values are examined, StereoLabs has lower values for right elbow angle flexion/extension, left elbow angle flexion/extension.

**Table 2:** Reliability between methods w.r.t. golden standard for elbow.

Motion	Metric	StereoLabs [Multi]	StereoLabs [Single]	MediaPipe [2D]	MediaPipe [3D]	YOLO pose [8n]	YOLO pose [8x-p6]
REFE	Bias	−3.47	−0.92	−3.87	10.13	−14.386	−3.74
	LoA	−34.52 to 27.59	−16.87 to 14.89	−44.24 to 36.51	−61.89 to 82.16	−59.85 to 31.07	−44.42 to 36.94
	ICC	0.97	0.99	0.95	0.61	0.89	0.95
LEFE	Bias	6.44	20.45	−1.54	34.50	−32.47	−0.58
	LoA	−30.49 to 43.36	−34.16 to 75.05	−45.43 to 42.35	−45.01 to 114.00	−106.71 to 41.78	−45.12 to 43.95
	ICC	0.96	0.86	0.94	0.42	0.57	0.94

REFE, Right Elbow Flexion/Extension; LEFE, Left Elbow Flexion/Extension.

In the hip comparison, StereoLabs has higher ICC values for flexion/extension of the right hip (StereoLabs [Multi]: 0.91, StereoLabs [Single]: 0.90, MediaPipe [2D]: 0.47, MediaPipe [3D]: 0.89, YOLO Pose [8n]: −0.031, YOLO Pose [8x-p6]: 0.44). MediaPipe [2D], YOLO Pose and YOLO Pose [8x-p6] have higher ICC values for right hip abduction/adduction (StereoLabs [Multi]: 0.86, StereoLabs [Single]: 0.88, MediaPipe [2D]: 0.94, MediaPipe [3D]: 0.43, YOLO Pose [8n]: 0.94, YOLO Pose [8x-p6]: 0.94). StereoLabs [Single] and YOLO Pose [8x-p6] have higher ICC values for right hip rotation (StereoLabs [Multi]: 0.54, StereoLabs [Single]: 0.93, MediaPipe [2D]: 0.94, MediaPipe [3D]: 0.27, YOLO Pose [8n]: 0.52, YOLO Pose [8x-p6]: 0.93). MediaPipe [2D] and YOLO Pose [8x-p6] have higher ICC values for left hip rotation (StereoLabs [Multi]: 0.71, StereoLabs [Single]:0.88, MediaPipe [2D]: 0.95, MediaPipe [3D]: 0.32, YOLO Pose [8n]: 0.84, YOLO Pose [8x-p6]: 0.94). StereoLabs [Single] and MediaPipe [3D] have higher ICC values for left hip flexion/extension (StereoLabs [Multi]: 0.81, StereoLabs [Single]:0.96, MediaPipe [2D]: 0.81, MediaPipe [3D]: 0.93, YOLO Pose [8n]: 0.20, YOLO Pose [8x-p6]: 0.83). MediaPipe [2D] has higher ICC values for left hip abduction/adduction (StereoLabs [Multi]: 0.80, StereoLabs [Single]: 0.79, MediaPipe [2D]: 0.90, MediaPipe [3D]: 0.46, YOLO Pose [8n]: 0.69, YOLO Pose [8x-p6]: 0.2 (Table 3).

**Table 3:** Reliability between methods w.r.t. golden standard for hip.

Motion	Metric	StereoLabs [Multi]	StereoLabs [Single]	MediaPipe [2D]	MediaPipe [3D]	YOLO pose [8n]	YOLO pose [8x-p6]
RHFE	Bias	14.34	12.53	35.32	−10.22	50.39	39.31
	LoA	−6.16 to 34.83	−18.192 to 43.25	−12.08 to 82.71	−47.12 to 26.69	−7.51 to 108.29	3.27 to 75.36
	ICC	0.91	0.90	0.47	0.89	−0.03	0.44
RHAA	Bias	−8.21	−3.75	2.13	8.99	−3.45	−2.42
	LoA	−33.42 to 16.99	−30.39 to 22.89	−10.15 to 14.41	−14.86 to 32.83	−15.48 to 8.59	−14.92 to 10.09
	ICC	0.86	0.88	0.94	0.43	0.94	0.94
RHR	Bias	−0.52	4.80	2.64	−26.46	9.81	0.01
	LoA	−23.29 to 22.24	−14.42 to 24.01	−17.78 to 23.06	−66.11 to 13.20	−27.98 to 47.59	−23.05 to 23.06
	ICC	0.91	0.93	0.94	0.27	0.52	0.93
LHFE	Bias	10.46	0.59	15.08	−12.87	28.44	14.56
	LoA	−31.33 to 52.24	−24.42 to 25.60	−23.29 to 53.45	−43.55 to 17.80	−35.26 to 92.13	−21.56 to 50.67
	ICC	0.81	0.96	0.81	0.93	0.20	0.83
LHAA	Bias	−10.08	−12.47	−5.450	10.29	9.12	15.33
	LoA	−35.03 to 14.87	−34.50 to 9.56	−18.68 to 7.78	−15.14 to 35.72	−4.89 to 23.13	−6.07 to 36.73
	ICC	0.80	0.79	0.9	0.46	0.69	0.2
LHR	Bias	−7.78	−8.66	2.03	−32.53	−1.07	4.15
	LoA	−39.13 to 23.56	−29.59 to 12.28	−18.36 to 22.42	−66.77 to 1.71	−28.59 to 26.45	−17.29 to 25.59
	ICC	0.71	0.88	0.95	0.32	0.84	0.94

RHFE, Right Hip Flexion/Extension; RHAA, Right Hip Abduction/Adduction; RHR, Right Hip Rotation; LHFE, Left Hip Flexion/Extension; LHAA, Left Hip Abduction/Adduction; LHR, Left Hip Rotation.

In the comparison made for the knee, StereoLabs has higher ICC values for right knee angle flexion/extension (StereoLabs [Multi]: 0.95, StereoLabs [Single]: 0.24, MediaPipe [2D]: 0.63, MediaPipe [3D]: 0.76, YOLO Pose [8n]: −0.03, YOLO Pose [8x-p6]: 0.22) and left knee angle flexion/extension (StereoLabs [Multi]: 0.95, StereoLabs [Single]: 0.91, MediaPipe [2D]: 0.57, MediaPipe [3D]: 0.71, YOLO Pose [8n]: 0.18, YOLO Pose [8x-p6]: 0.49) (Table 4). In addition, when the MDC and SEM values are examined, StereoLabs has lower values for right knee angle flexion/extension and left knee angle flexion/extension. However, notably low ICC values (e.g., YOLOv8n ICC = −0.03 for right knee flexion/extension) may result from occlusions, insufficient depth perception in single-camera setups, or pose estimation errors. Such discrepancies are more pronounced when movements involve overlapping limbs.

**Table 4:** Reliability between methods w.r.t. golden standard for knee.

Motion	Metric	StereoLabs [Multi]	StereoLabs [Single]	MediaPipe [2D]	MediaPipe [3D]	YOLO pose [8n]	YOLO pose [8x-p6]
RKFE	Bias	3.68	43.25	32.19	−23.10	64.05	49.65
	LoA	−32.26 to 39.60	−34.03 to 120.52	−20.62 to 84.99	−72.55 to 26.35	−17.37 to 145.46	−19.99 to 119.29
	ICC	0.95	0.24	0.63	0.76	−0.03	0.22
LKFE	Bias	2.93	−1.52	36.80	−28.08	58.42	38.57
	LoA	−31.99 to 37.84	−42.94 to 39.91	−10.91 to 84.51	−74.24 to 18.09	−5.60 to 122.44	−13.89 to 91.03
	ICC	0.95	0.91	0.57	0.71	0.18	0.49

RKFE, Right Knee Flexion/Extension; LKFE, Left Knee Flexion/Extension.

**Figure 3:**
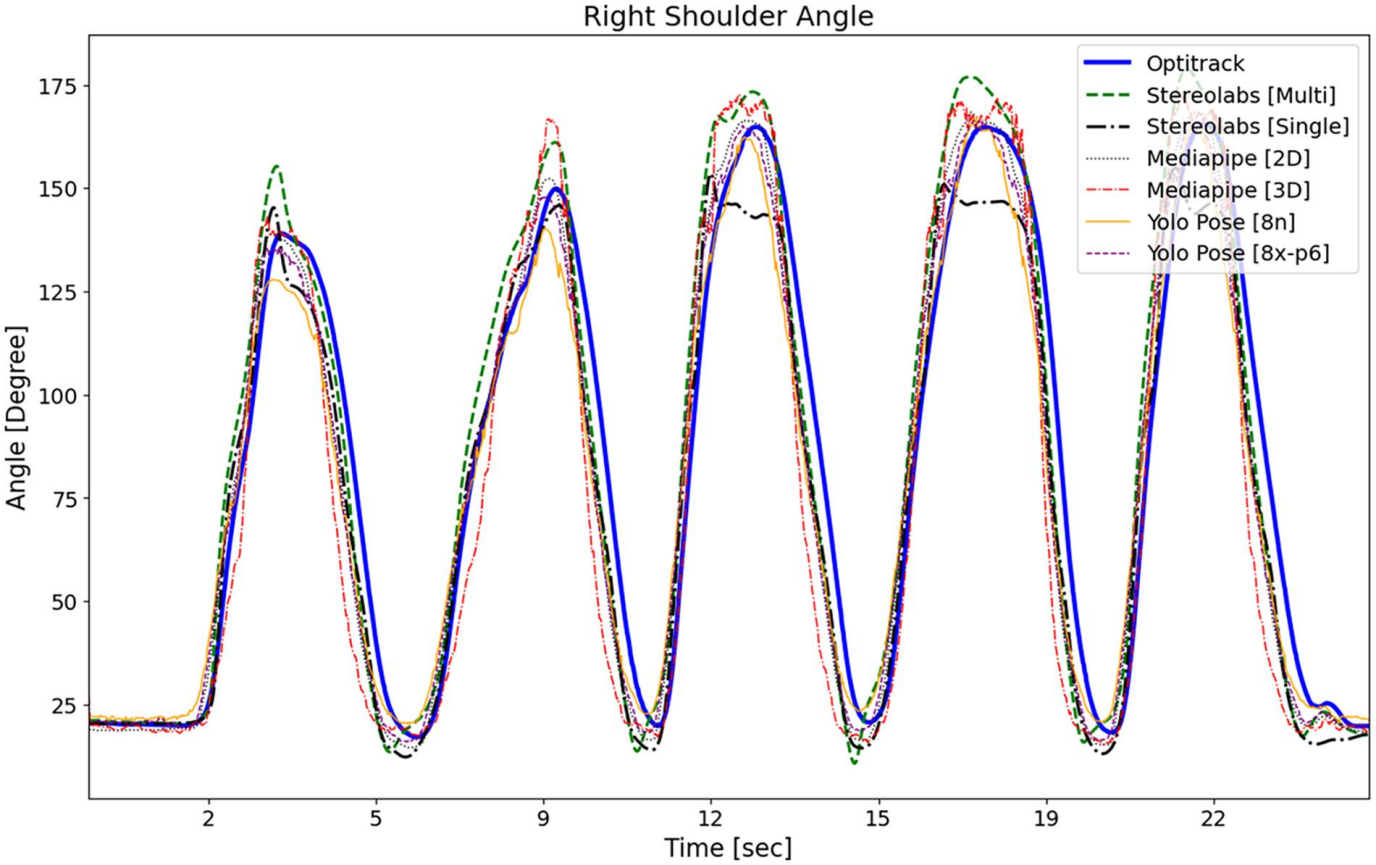
Right shoulder abduction/adduction angle.

**Figure 4:**
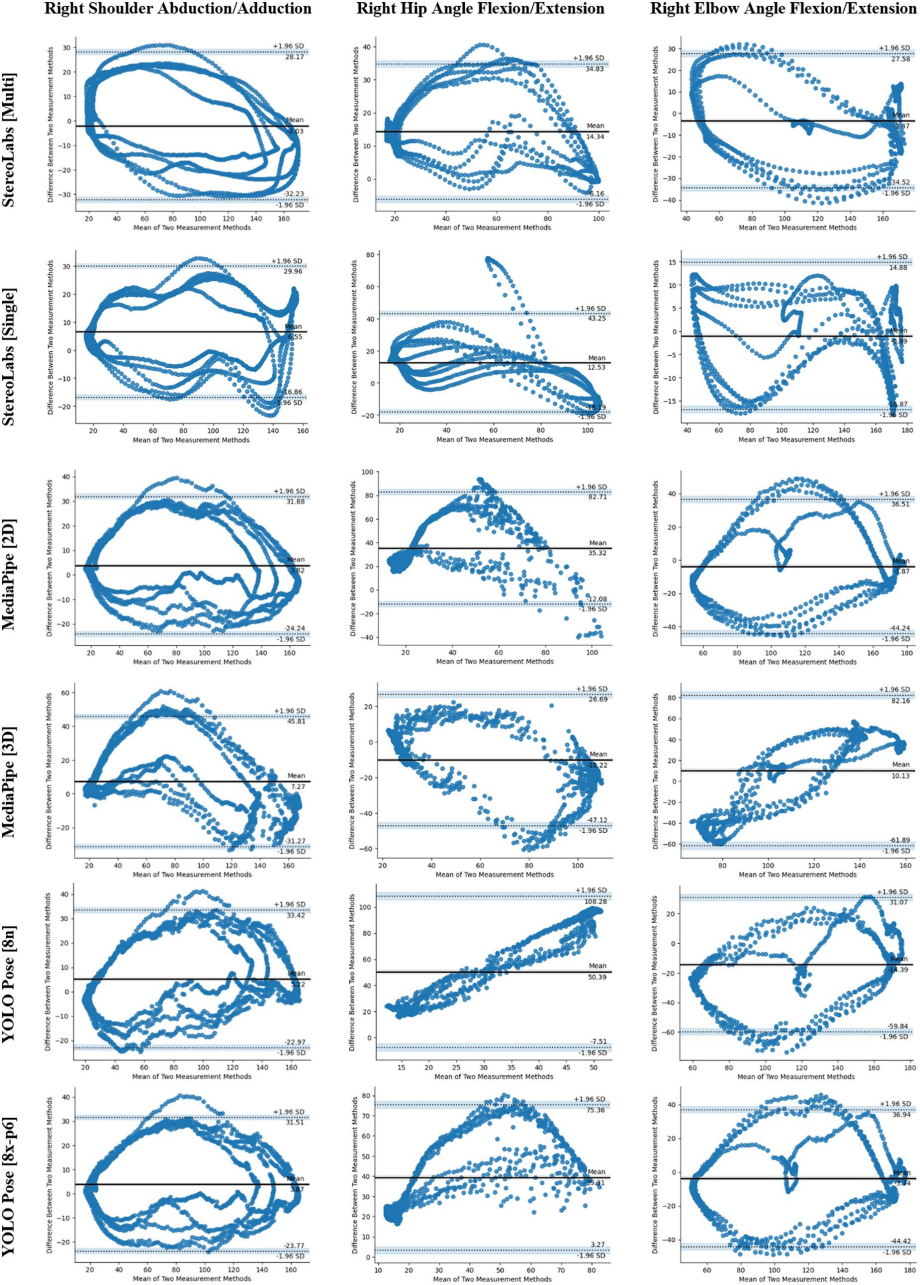
Sample Bland-Altman plots showing bias and LoA of markerless methods vs. OptiTrack.

The obtained measurements were plotted against time to compare with the gold standard. Figure 3 shows the right shoulder abduction/adduction angle over time for different methods/models (Angle graphs of other joints are given in the Supplementary Material.). The measurements obtained were also tested with the Bland-Altman method to compare different methods with the gold standard. Sample Bland-Altman plots with bias value and 95% confidence interval (LoA) are given in Figure 4. The row labels indicate the methods, and the column labels indicate the motion shown in each subplot. Each subplot corresponds to a specific motion-method pair, where each dot represents a paired measurement comparing the markerless method with the OptiTrack gold standard. The solid horizontal line denotes the mean bias, and the dashed lines indicate the upper and lower LoA. As can be seen in the figure, most of the measurements are within the LOA interval. Also, at the extreme points of the movement, the amount of error approaches zero. In our interpretation, the mean deviation from zero indicates the level of systematic bias, while the distance between upper and lower limits represents the degree of reliability. Outlier rates were quantified and were generally within the threshold of 5% or less than reported in the literature. Angle over time plots and Bland-Altman plots for other movements can be found in the Supplementary Material.

## 4 Discussion

For studies where accurate measurement of body angles and extremities is critical, determining the most successful measurement method is of great importance. The accuracy of the measurement directly affects the success of the study. Accordingly, the most successful system, whether marker-based or markerless, should be selected based on the type of study. In this study, various markerless systems were compared with a marker-based system. The OptiTrack system was considered the gold standard among marker-based systems, while markerless systems were evaluated using both multi-camera and single-stereo-camera solutions, along with 2D and 3D methods. In the markerless category, StereoLabs’ multi-camera and single-camera systems, along with single-camera methods from YOLOv8 Pose and both 2D and 3D MediaPipe methods, are utilized. To our knowledge, this study presents one of the most comprehensive comparisons involving this specific combination of depth-based and RGB-only architectures across both upper and lower extremity movements. Unlike many previous studies that mainly focused on gait or on either lower- or upper-limb kinematics, our analysis encompasses both upper and lower extremity movements, providing a more comprehensive assessment of system reliability for diverse functional tasks. In this respect, our work complements earlier validation studies on gait and daily activities [e.g., [12, 17]] by extending the comparison of markerless and marker-based systems to simple upper-limb and shoulder-dominated motions.

According to the results obtained from the study, the multi-camera StereoLabs system showed the highest relative reliability among the markerless systems in terms of ICC (average: 0.915) and generally smaller biases across several joint angles. Single-camera methods from both StereoLabs and MediaPipe, as well as the YOLOv8 Pose models, yielded lower ICC values and larger biases overall, but still demonstrated moderate agreement for some movements under the controlled, low-velocity conditions tested here. In particular, MediaPipe 2D (average ICC: 0.848) and the single-camera StereoLabs configuration (average ICC: 0.868) showed relative reliability that was closer to that of the multi-camera StereoLabs model, whereas MediaPipe 3D (average ICC: 0.658), YOLO Pose 8n (average ICC: 0.613), and YOLO Pose 8x-p6 (average ICC: 0.740) exhibited more variable performance across joints and movements. This general pattern is consistent with previous work comparing depth-camera–based markerless systems with a gold-standard marker-based reference. For example, Özsoy et al. reported that both Kinect v2 and Azure Kinect showed reasonably high reliability for shoulder flexion and abduction, but substantially lower ICCs for rotation movements when compared with a marker-based system, indicating that agreement can deteriorate markedly for more challenging joint configurations [21]. Taken together with our findings, these results suggest that depth-based and learning-based markerless systems can achieve moderate-to-high relative reliability for selected movements, but that their absolute agreement with marker-based motion capture remains limited, especially for rotations and conditions with substantial occlusion.

While ICC values provide insight into the correlation between systems, the practical applicability must be assessed through absolute error metrics (SEM, MDC) and Bland-Altman analysis (LoA). Our results reveal that despite high average ICC values for some systems, the magnitude of error reached clinically unacceptable levels in specific instances. For example, MediaPipe [3D] exhibited extremely wide LoA for RSHAA (approx. $140^{\circ }$) and a substantial MDC for LEFE ($85.6^{\circ }$). Such large errors indicate that the system cannot reliably detect meaningful changes in joint angles, rendering it impractical for clinical or sports science applications where precision is important and errors on the order of only a few degrees (often < $5^{\circ }$) are typically considered acceptable. From a biomechanical perspective, joint angle errors of $10^{\circ }$–$30^{\circ }$ such as those observed in some conditions in this study can substantially affect downstream estimates of joint moments, muscle forces, and tissue loading obtained from inverse dynamics and musculoskeletal models, and may therefore compromise tissue-level metrics and injury-risk predictions [31]. Furthermore, the occurrence of negative or near-zero ICC values (e.g., YOLOv8 [8n] for right hip and knee flexion/extension) signifies a systematic failure of the model to track these movements. These failures are likely attributable to the inherent limitations of single-camera setups in resolving depth ambiguity and handling occlusions, particularly during movements where limb segments overlap significantly relative to the camera viewpoint.

A notable and counter-intuitive finding was the superior performance of MediaPipe [2D] (Avg. ICC: 0.848) compared to MediaPipe [3D] (Avg. ICC: 0.658). Although 3D models aim to provide depth information, the process of estimating 3D poses from a single RGB image is inherently challenging (an ill-posed problem). Errors in depth estimation can propagate into the calculation of joint angles. This is particularly relevant for the simple, largely planar movements assessed in this study, where the 2D model may benefit from robust keypoint localization on the image plane without the added complexity and potential error of 3D reconstruction.

The performance discrepancies observed between systems can be largely attributed to their underlying architectural differences and depth estimation strategies. The StereoLabs system utilizes stereoscopic depth sensing, directly resolving the depth ambiguity that monocular RGB-based models must estimate algorithmically. For single-camera models like MediaPipe and YOLOv8, the lifting of 2D keypoints to 3D space represents an ill-posed problem; lacking direct depth data, these models rely heavily on training datasets, which may not generalize well to specific limb occlusions or the rotational tasks performed in this study. Furthermore, the varying keypoint detection strategies and the absence of temporal consistency constraints in standard frame-by-frame inference contribute to the higher variability observed in the markerless methods compared to the multi-camera fusion of the gold standard.

In upper extremity movements, the StereoLabs multi-camera and single-camera systems have been observed to showed higher ICC values and smaller errors, particularly in rotation movements where the multi-camera system has captured rotation much more successfully compared to other systems. The reason rotations are more accurately determined by the multi-camera system is thought to be that it is easier to see the rotations from different angles with all cameras involved. In general, single-camera systems such as MediaPipe 2D, YOLO Pose [8x-p6], and StereoLabs’ single-camera solution provided more reliable results in the hip area compared to the multi-camera and single-camera system of StereoLabs. However, MediaPipe 3D stands as an exception in terms of its performance, with notably lower reliability as measured by ICC values across almost all hip-related motions (except right hip flexion/extension). It is thought that the detection of the hip bone with the multi-camera system failed due to the uniform color of the clothing and the lack of agreement among all cameras, which affected the results. In single-camera systems, the system can more easily predict the joint point from a fixed front position only.

Regarding the knee area, the multi-camera system again showed the relatively closest agreement with the gold-standard system among the markerless options, although notable discrepancies were still present for some movements. Among the single-camera systems, MediaPipe’s 3D model displayed somewhat better agreement than its 2D counterpart under the present testing conditions. A plausible explanation is that the 2D model, lacking depth cues, may be more susceptible to errors in situations where limb overlap occurs. These observations suggest that access to depth information may contribute to improved tracking performance for joints prone to occlusion, although further multi-subject evaluations would be needed to confirm this pattern more robustly.

Although marker-based systems are considered the gold standard for quantitative motion analysis, the experimental configuration required to obtain marker-based data may influence the performance of the markerless pipelines. In this study, the participant wore a tight black bodysuit with reflective markers to enable accurate tracking with the OptiTrack system. The markers themselves cover only a small portion of the body surface, but the combination of a uniform dark outfit and bright reflective spheres may alter local texture and contrast around anatomical landmarks in the RGB and RGB–D images, particularly for single-camera views without depth information. As a result, the agreement metrics reported here may slightly underestimate the performance that markerless systems could achieve in markerless-only recordings with more varied clothing. At the same time, our setup reflects a realistic scenario in which marker-based and markerless systems are used concurrently and therefore provides a conservative estimate of their relative performance under suboptimal optical conditions.

Furthermore, the study primarily evaluated simple movements at a camera speed of 60 Hz, which is sufficient for slow, simple actions but inadequate for capturing high-speed movements. As all models were tied to the same 60 Hz camera, a general decline in performance for faster movements is likely. Therefore, for studies involving high-speed movements, the use of a camera with a higher frame rate will be necessary. Moreover, multi-camera methods are anticipated to become increasingly important for accurately capturing complex joint movements.

## 5 Limitations and future work

This study provides a comprehensive comparative analysis of markerless technologies; however, several limitations must be acknowledged. Foremost, the study employed an intensive SSED. As detailed in the Methods section, this design was intentionally selected to eliminate inter-subject variability, thereby maximizing internal validity and isolating the inherent measurement variance of the different technologies. Although the use of a single subject restricts the generalization of findings across different genders, body types, and ages, this design was deliberately chosen to minimize confounding variables. By strictly controlling for inter-subject variability, the inherent instrument noise and algorithmic error of each system were isolated, establishing a necessary baseline for technical precision before large-scale population studies. The results should be interpreted as a foundational proof-of-concept comparison rather than a definitive assessment of ecological validity. This study was designed as a preliminary in vivo proof-of-concept, focusing on controlled, single-DoF movements performed repeatedly by the same participant to ensure that any observed differences were attributable to the systems rather than inter-individual variability. While this design limits generalizability, it provides a consistent baseline for future multi-participant, multi-condition studies.

Furthermore, the study focused on simple, isolated upper and lower limb movements performed at relatively low velocities. These fundamental movements were selected to establish baseline accuracy without the analytical complexity associated with high-speed dynamics or frequent occlusions. Validating these elementary, single-plane motions is considered a necessary prerequisite, as systems failing to achieve high reliability under these controlled conditions would be inherently unsuitable for complex, multi-joint sporting tasks.

Another limitation relates to the clothing configuration used in this study. The participant wore a standardised black bodysuit with reflective markers to enable accurate tracking with the OptiTrack reference system. While this outfit is appropriate for marker-based tracking, it does not necessarily represent the optimal visual conditions for the tested markerless pipelines and is likely to have influenced their absolute accuracy.

Future research must build upon this foundational comparison by validating these technologies with a larger and more diverse participant cohort to establish population-level reliability. Additionally, future studies must investigate the performance of these systems during dynamic, high-speed, and ecologically valid movements, utilizing higher acquisition frequencies.

## 6 Conclusion

The significance of this work is in its comprehensive comparison of marker-based vs. markerless motion capture systems, emphasizing both upper and lower extremities. The results provide significant insights into system reliability and practicability, informing the choice of the most appropriate technology for diverse applications according to particular requirements, movement complexities, and actual performance.

The OptiTrack system, dependent on markers, is regarded as the gold standard in its domain. The StereoLabs system uses many cameras and operates without markers, showing remarkable performance with high ICC values (average ICC [Multi]: 0.915, average ICC [Single]: 0868). This makes it a viable choice when marker installation is impracticable. Based on our findings, the multi-camera StereoLabs configuration generally showed higher ICC values and smaller biases than the other markerless pipelines for several joint angles. However, the corresponding limits of agreement remained wide (up to approximately $30^{\circ }$ in some conditions), indicating that it should be viewed as the comparatively best-performing markerless option in this study rather than a high-accuracy replacement for marker-based motion capture in clinical or sports biomechanics. Single-camera solutions such as MediaPipe 2D offer accessible, cost-effective options for rehabilitation monitoring or telehealth applications, while YOLOv8 models may be preferred for real-time sports analysis where speed is prioritized over maximal accuracy. MediaPipe [2D] (average ICC: 0.848) and YOLO Pose [8x-p6] (average ICC: 0.740), both single-camera systems, have exhibited satisfactory precision for less complicated motions.

Although marker-based systems remain the gold standard methods for detailed analysis, computer vision and machine learning improvements have made markerless systems more suitable for various applications, particularly in cases where cost, ease, and non-invasiveness are essential considerations. Nevertheless, the choice of system should be influenced by the demands of the application, considering the compromises in terms of precision, intricacy, and feasibility.

To bridge the gap between current capabilities and the rigorous demands of clinical and athletic applications, a clearer development roadmap is essential. Future advancements should prioritize the integration of multi-view fusion techniques to resolve single-camera occlusions and the incorporation of biomechanical constraints to prevent anatomically impossible poses. Furthermore, the utilization of larger training datasets and the establishment of cross-device standardization processes are critical for ensuring robustness across varying populations. Finally, integrating interpretability analysis (e.g., SHAP or LIME) into black-box deep learning models could provide valuable insights into failure modes, thereby increasing clinical trust in these emerging technologies.

## Data Availability

The processed data supporting the conclusions of this article will be made available by the authors, without undue reservation. The original camera images are not shared due to identifiable human features and associated ethical and privacy considerations. All processed point data from both the marker-based and markerless systems can be shared.
