# Supplementary material for: Reliability assessment of markerless technologies in biomechanical motion analysis: a performance comparison
Source: Front Sports Act Living. 2026 Jan 12;7:1712332. doi: 10.3389/fspor.2025.1712332 (PMC12833419; doi:10.3389/fspor.2025.1712332)
Supplement: Supplementary file 1 [file Datasheet1.pdf]

SUPPORTING MATERIALS

Reliability Assessment of Markerless Technologies in Biomechanical Motion Analysis: A Performance Comparison

Ibrahim Cem Balci, Irem Sayin, Serkan Salturk, Rana Gursoy, Umut Ozsoy, Husnu Caglar Dogru, Gokhan Akca, Ali Eraslan, Onurcan Sahin, Ali Anil Demircali, Huseyin Uvet

Contents:

Supplementary Figure 1. Right Shoulder Abduction/Adduction Bland-Altman Plots & Angle Values - Time Plot

Supplementary Figure 2. Right Shoulder Horizontal Abduction/Adduction Bland-Altman Plots & Angle Values - Time Plot

Supplementary Figure 3. Right Shoulder Rotation Bland-Altman Plots & Angle Values - Time Plot

Supplementary Figure 4. Left Shoulder Abduction/Adduction Bland-Altman Plots & Angle Values - Time Plot

Supplementary Figure 5. Left Shoulder Horizontal Abduction/Adduction Bland-Altman Plots & Angle Values - Time Plot

Supplementary Figure 6. Left Shoulder Rotation Bland-Altman Plots & Angle Values - Time Plot

Supplementary Figure 7. Right Elbow Angle Flexion/Extension Bland-Altman Plots & Angle Values - Time Plot

Supplementary Figure 8. Left Elbow Angle Flexion/Extension Bland-Altman Plots & Angle Values - Time Plot

Supplementary Figure 9. Right Hip Angle Flexion/Extension Bland-Altman Plots & Angle Values - Time Plot

Supplementary Figure 10. Right Hip Angle Abduction/Adduction Bland-Altman Plots & Angle Values - Time Plot

Supplementary Figure 11. Right Hip Angle Rotation Bland-Altman Plots & Angle Values - Time Plot

Supplementary Figure 12. Left Hip Angle Flexion/Extension Bland-Altman Plots & Angle Values - Time Plot

Supplementary Figure 13. Left Hip Angle Abduction/Adduction Bland-Altman Plots & Angle Values - Time Plot

Supplementary Figure 14. Left Hip Angle Rotation Bland-Altman Plots & Angle Values - Time Plot

Supplementary Figure 15. Right Knee Angle Flexion/Extension Bland-Altman Plots & Angle Values - Time Plot

Supplementary Figure 16. Left Knee Angle Flexion/Extension Bland-Altman Plots & Angle Values - Time Plot

Supplementary Table 1. Reliability between methods w.r.t. golden standard for shoulder

Supplementary Table 2. Reliability between methods w.r.t. golden standard for elbow

Supplementary Table 3. Reliability between methods w.r.t. golden standard for hip

Supplementary Table 4. Reliability between methods w.r.t. golden standard for knee

This supplementary material contains the complete set of Bland-Altman plots and Angle-Time plots for all examined joint angles, along with the tables containing the analysis results.

Supplementary Figures: The Bland-Altman plots compare each markerless system against the OptiTrack gold standard. In these plots, the title of each subplot indicates the markerless method being evaluated. The x-axis represents the mean of the two measurement methods (degrees), and the y-axis represents the difference (Markerless - OptiTrack) (degrees). Each dot represents a paired frame-by-frame measurement. The solid horizontal line denotes the mean bias, while the dashed lines indicate the upper and lower 95% Limits of Agreement (LoA). The Angle-Time plots illustrate the trajectory of the joint angles over time for all systems synchronously.

Supplementary Tables: Supplementary Tables 1-4 provide comprehensive statistical analysis results. While the tables presented in the main manuscript summarize the primary reliability metrics (Bias, LoA range, and Intraclass Correlation Coefficient [ICC]) to improve readability, these supplementary tables report the full set of calculated metrics, including the LoA Ratio, 95% Confidence Intervals (CI) for ICC, Standard Error of Measurement (SEM), and Minimal Detectable Change (MDC) for all comparisons.

Bland-Altman Plots

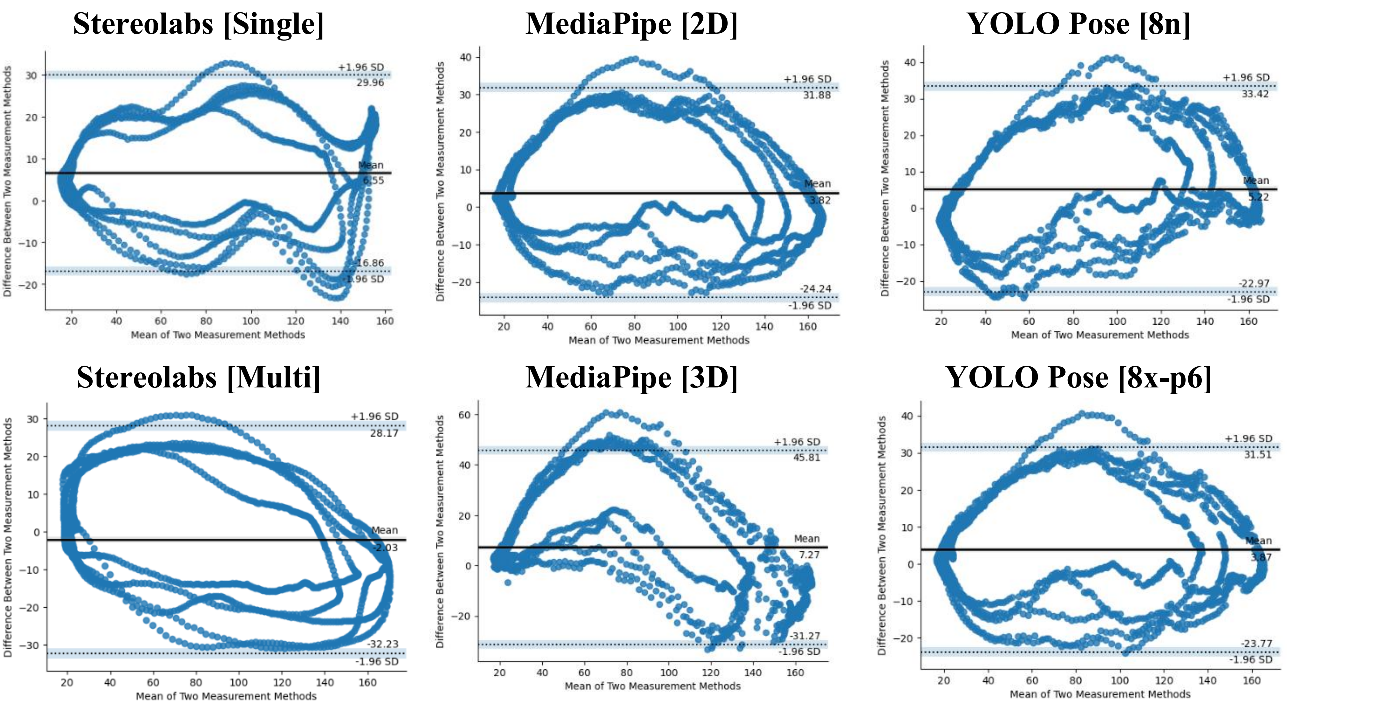

Angle Values - Time Plot

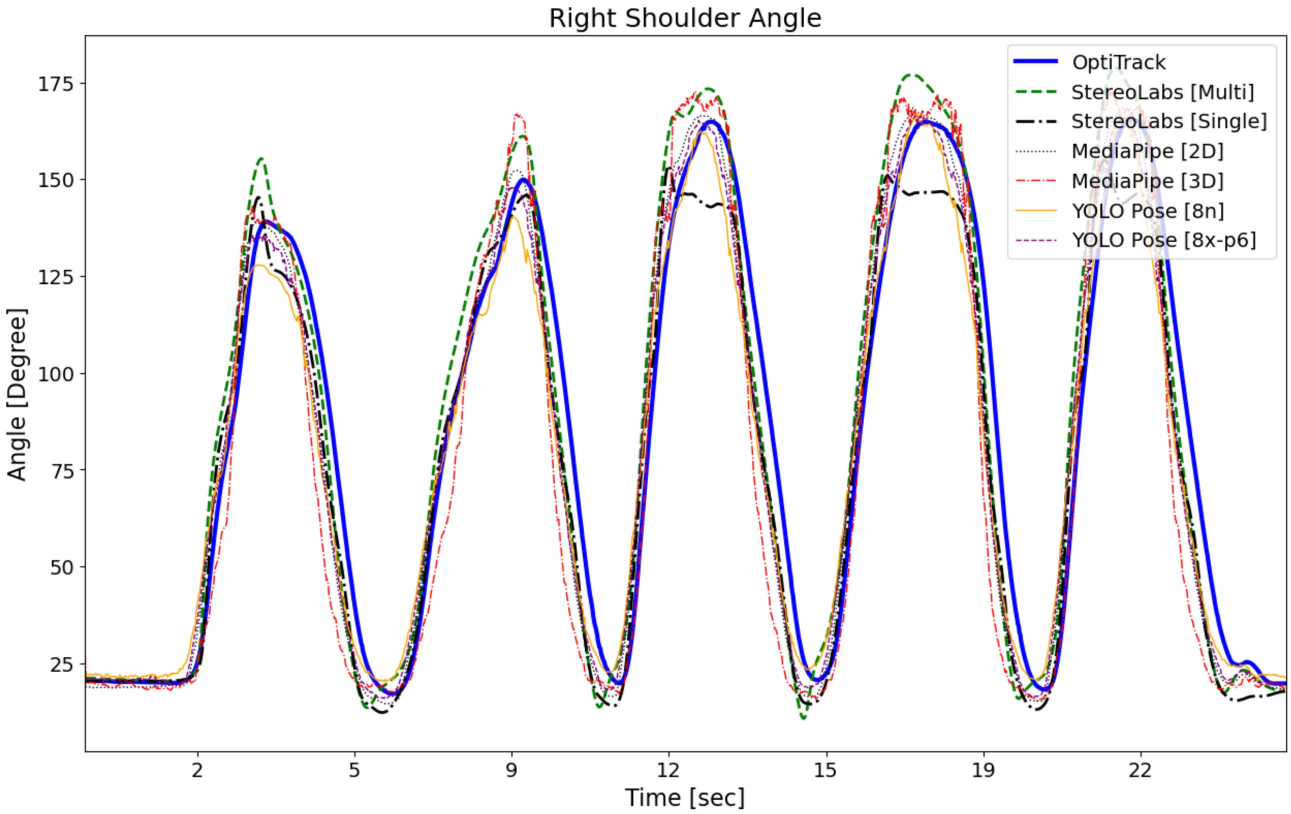

Supplementary Figure 1. Right Shoulder Abduction/Adduction Bland-Altman Plots & Angle Values - Time Plot

Bland-Altman Plots

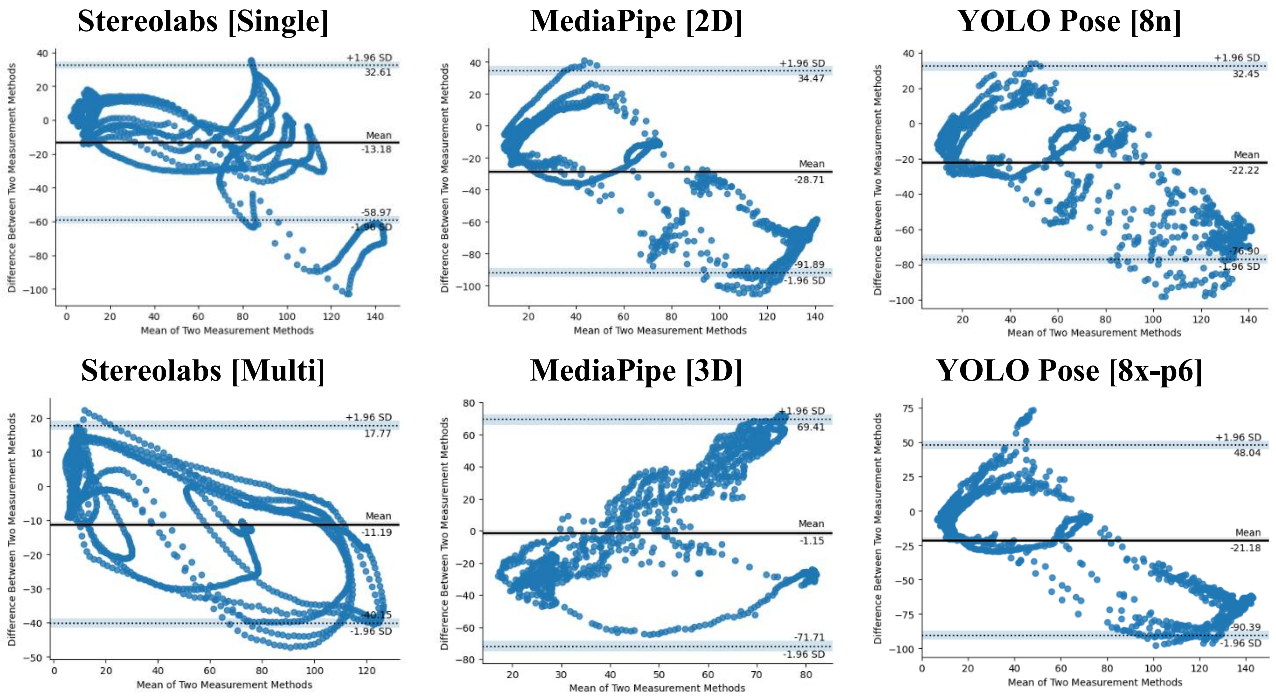

Angle Values - Time Plot

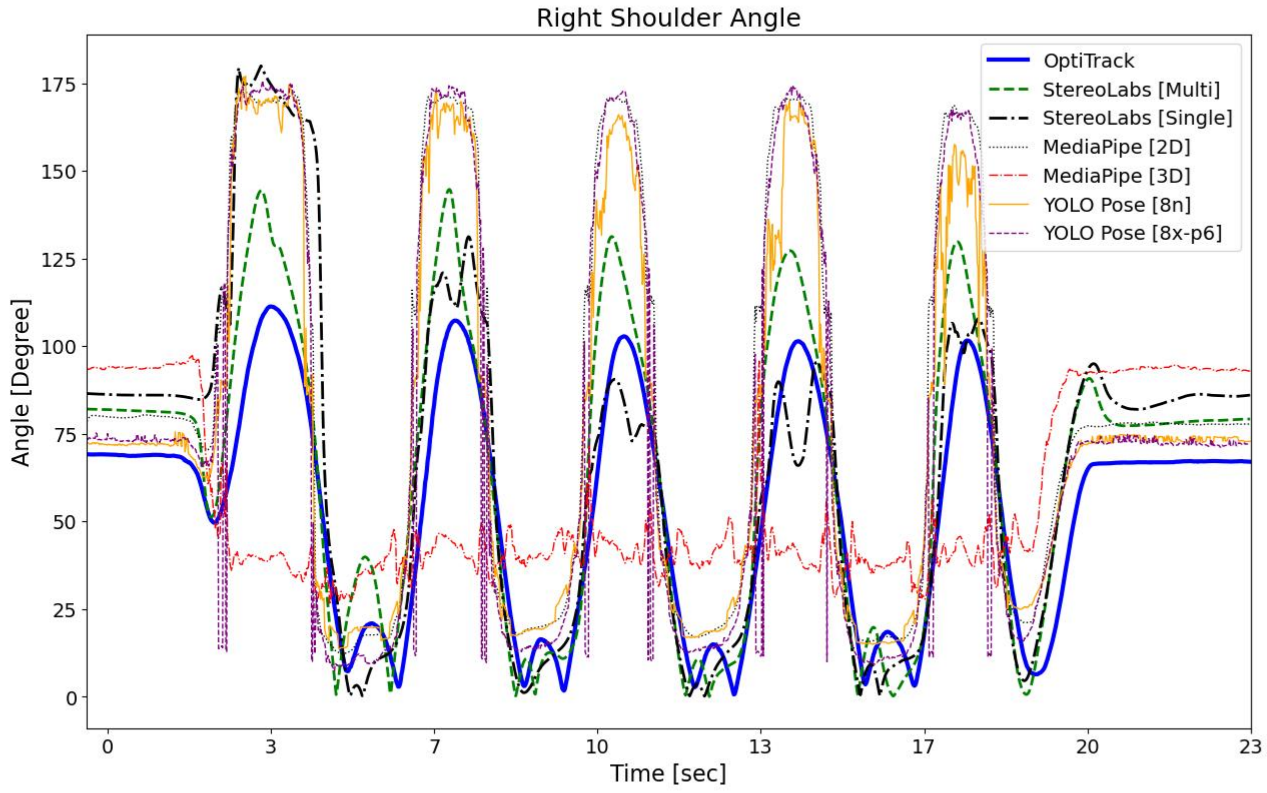

Supplementary Figure 2. Right Shoulder Horizontal Abduction/Adduction Bland-Altman Plots & Angle Values - Time Plot

Bland-Altman Plots

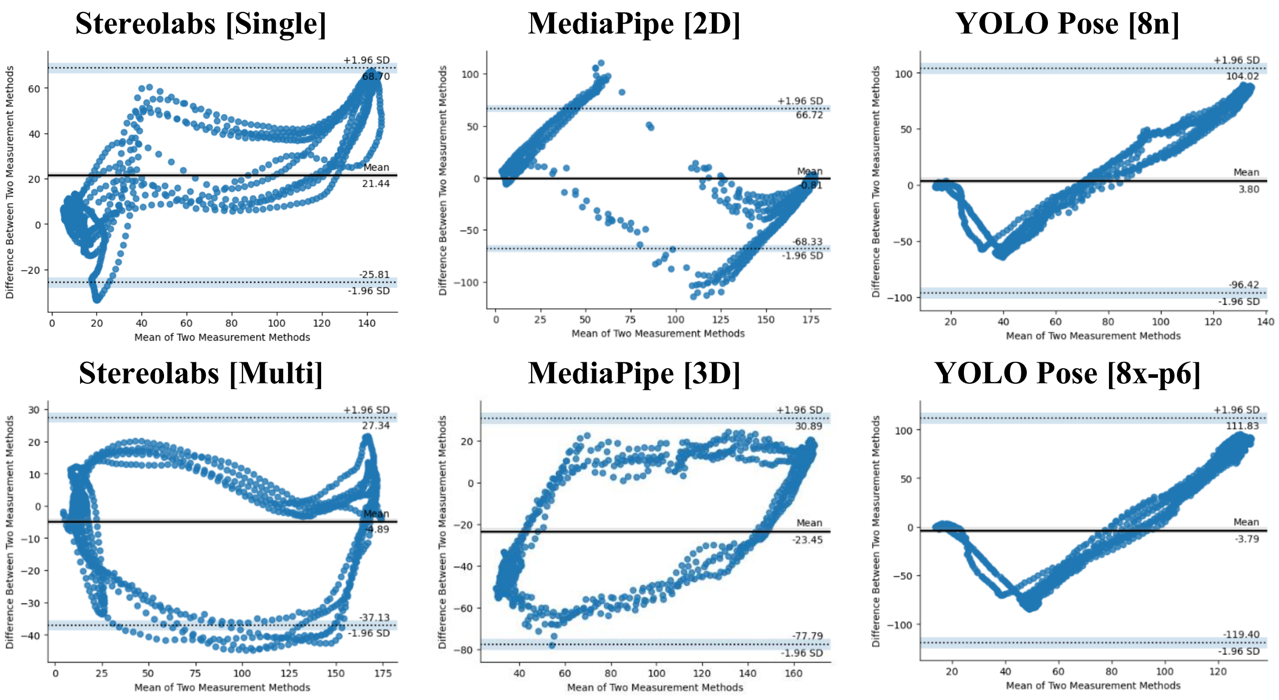

Angle Values - Time Plot

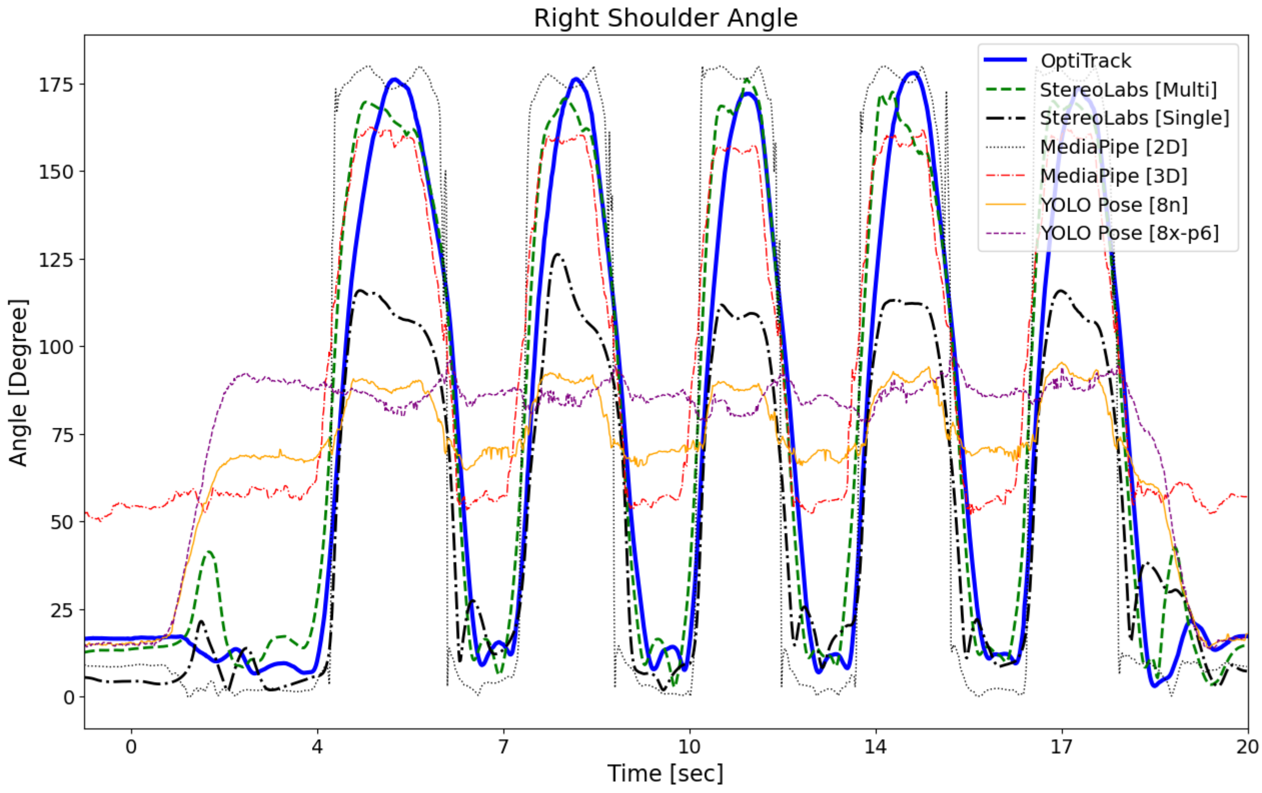

Supplementary Figure 3. Right Shoulder Rotation Bland-Altman Plots & Angle Values - Time Plot

Bland-Altman Plots

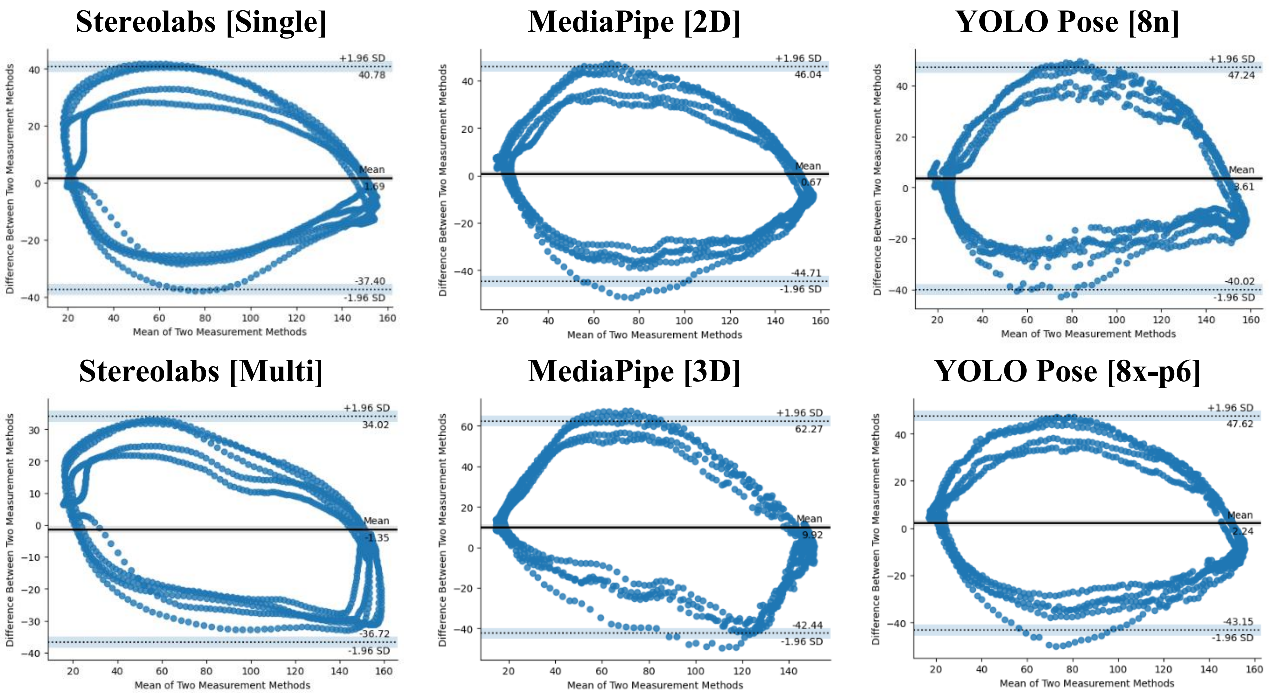

Angle Values - Time Plot

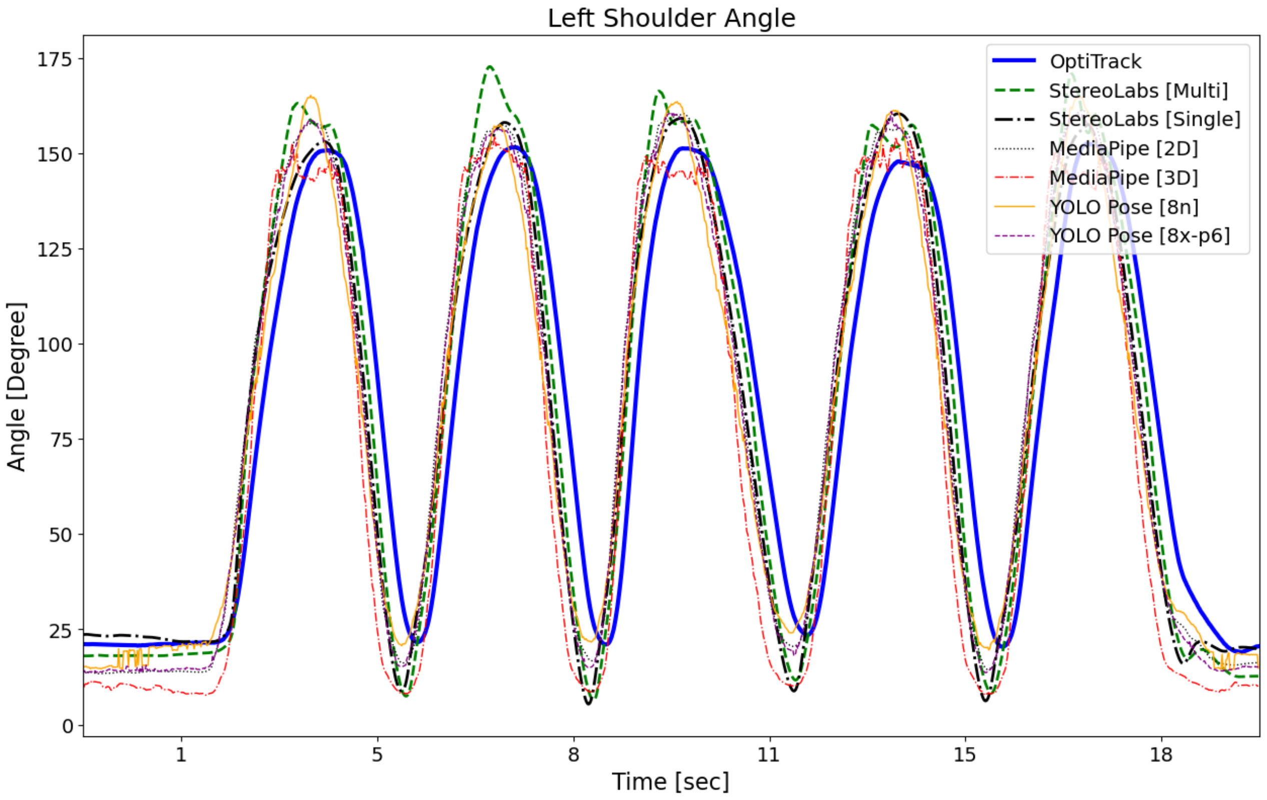

Supplementary Figure 4. Left Shoulder Abduction/Adduction Bland-Altman Plots & Angle Values - Time Plot

Bland-Altman Plots

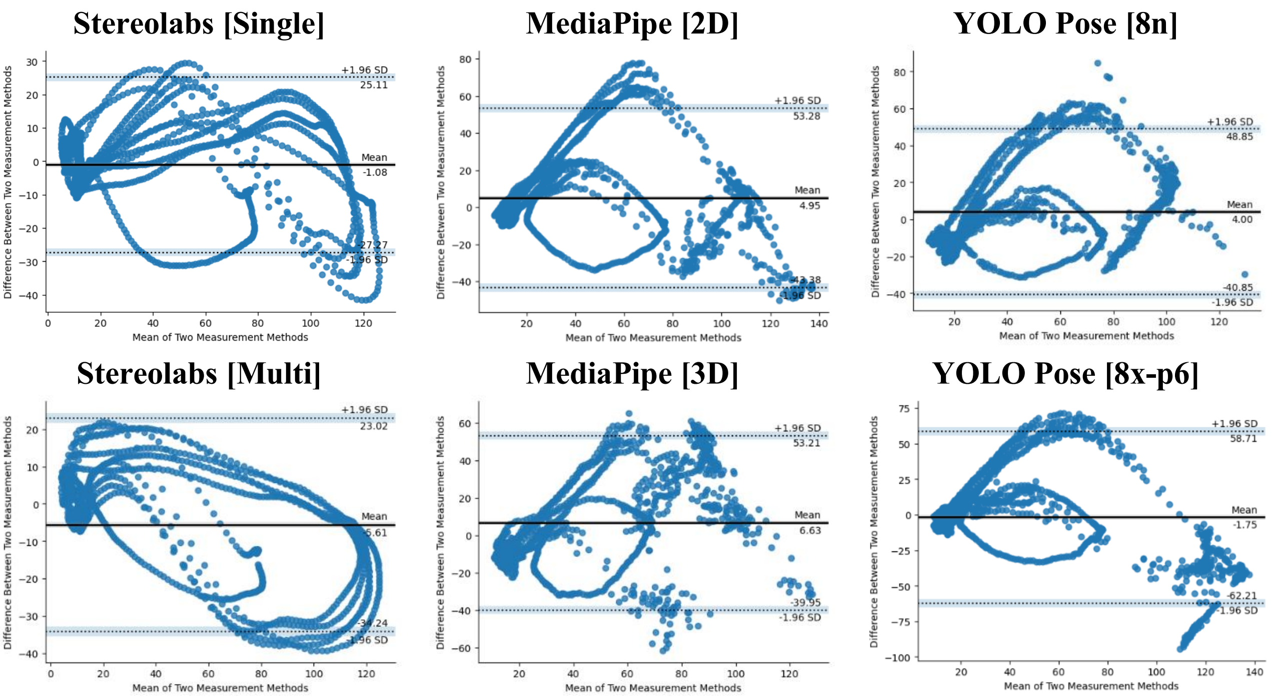

Angle Values - Time Plot

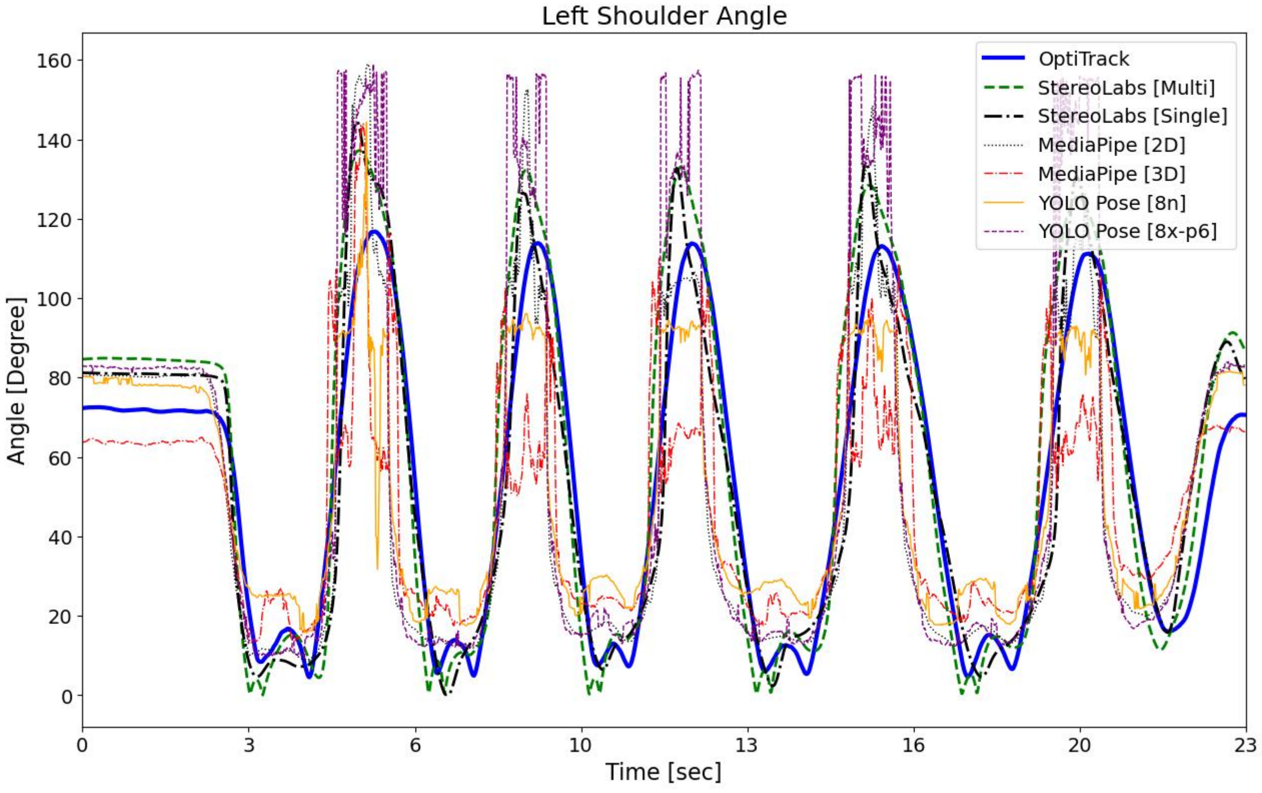

Supplementary Figure 5. Left Shoulder Horizontal Abduction/Adduction Bland-Altman Plots & Angle Values - Time Plot

Bland-Altman Plots

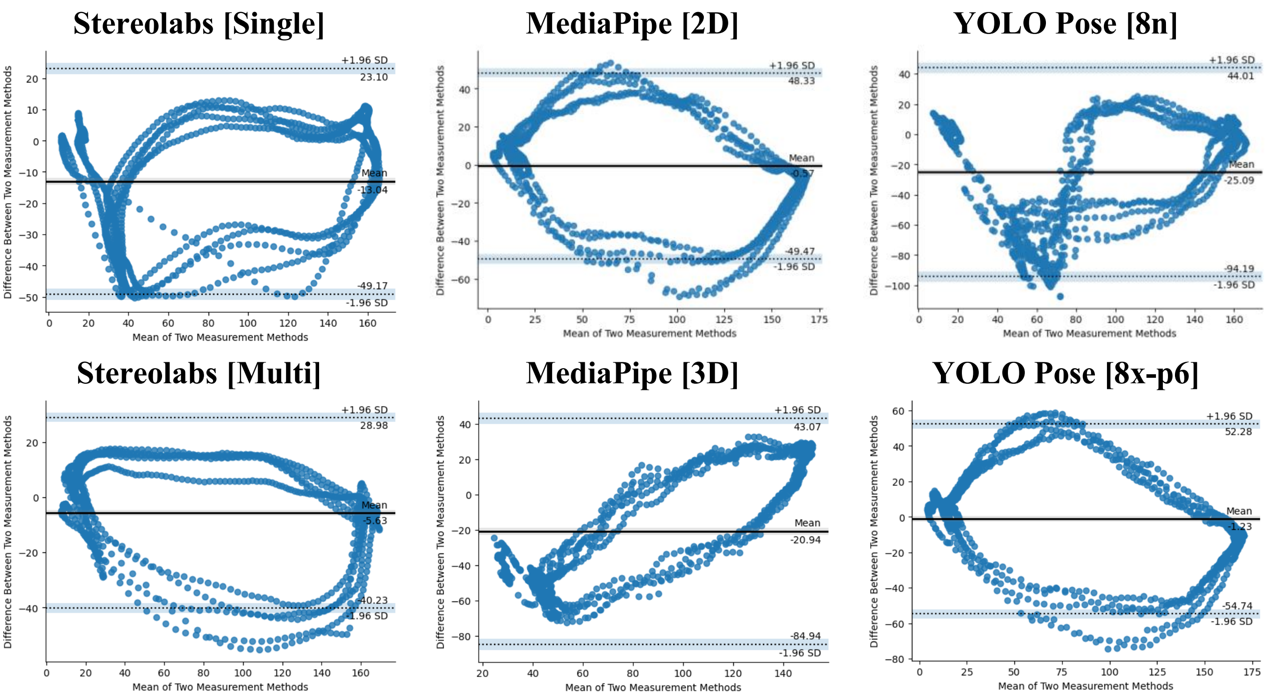

Angle Values - Time Plot

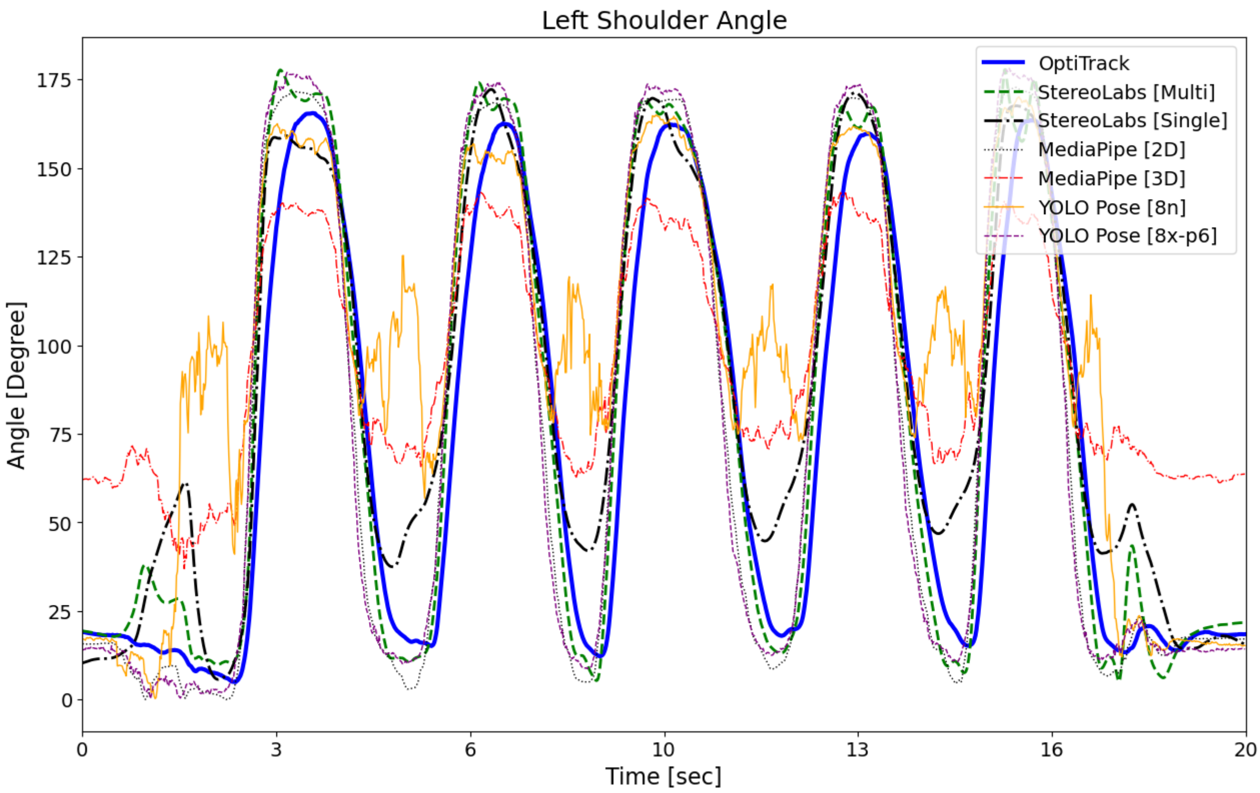

Supplementary Figure 6. Left Shoulder Rotation Bland-Altman Plots & Angle Values - Time Plot

Bland-Altman Plots

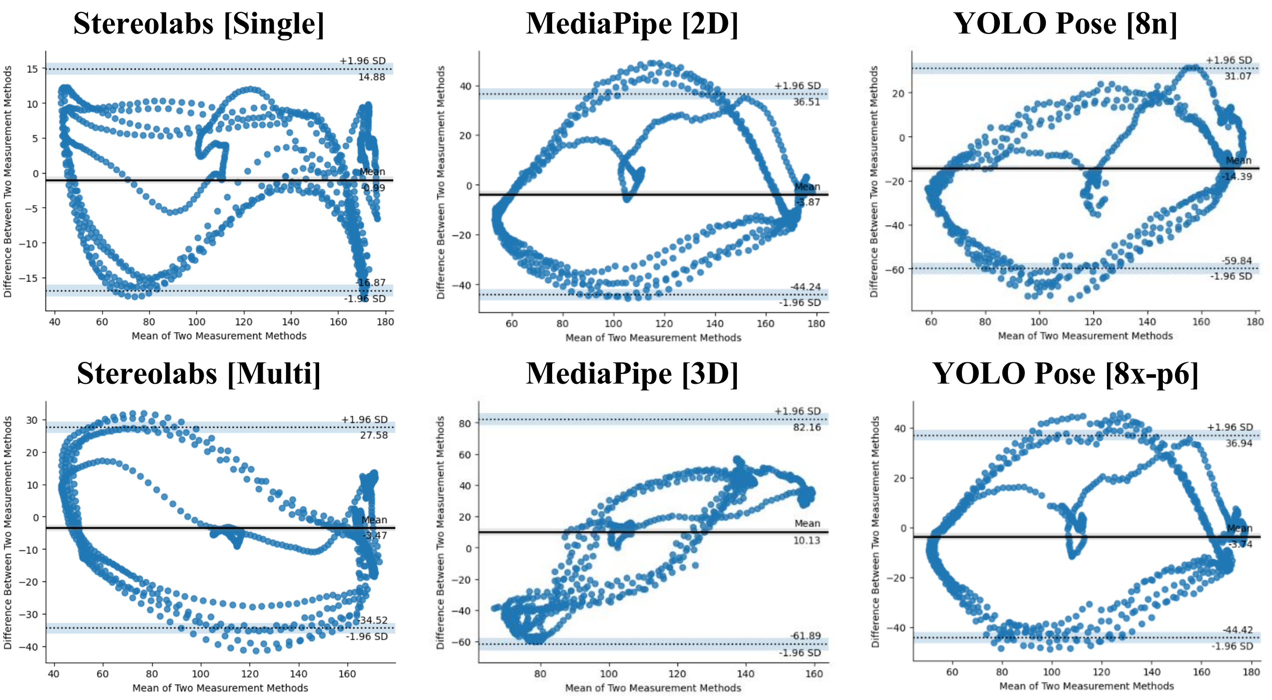

Angle Values - Time Plot

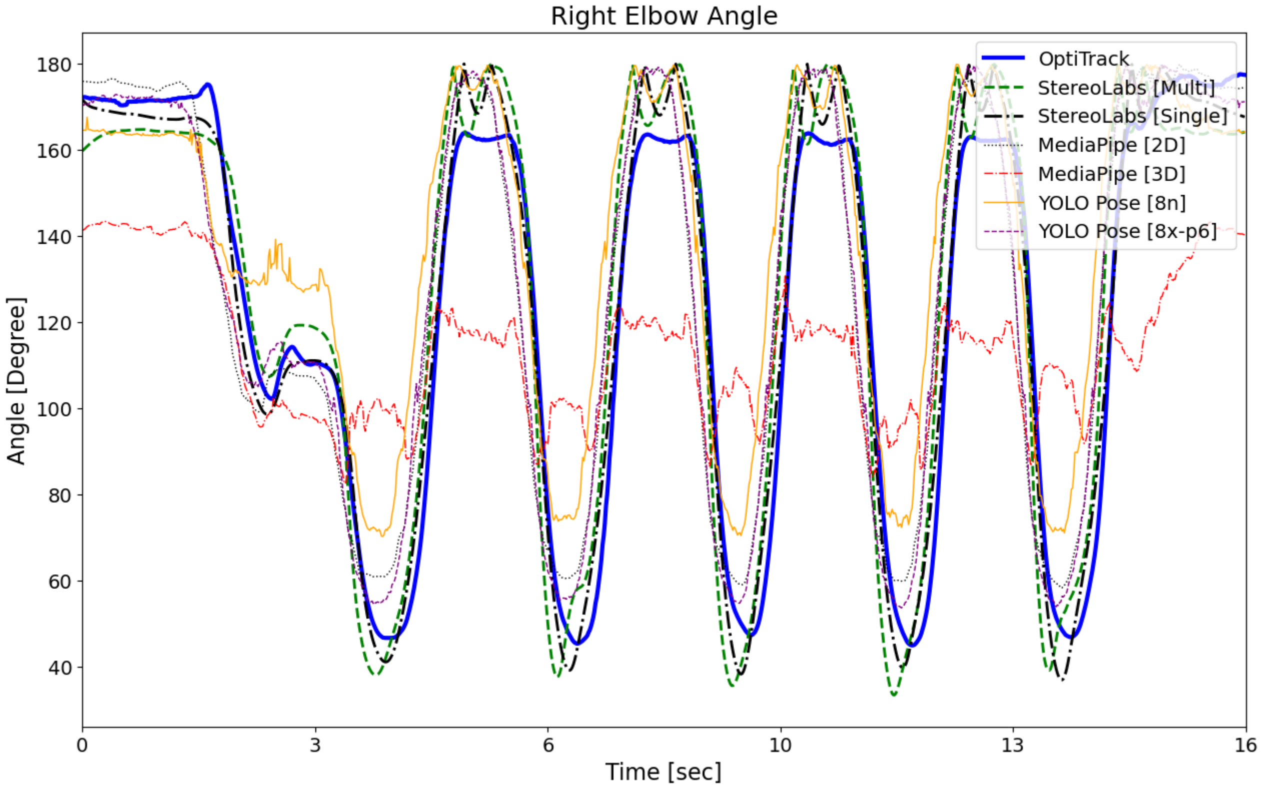

Supplementary Figure 7. Right Elbow Angle Flexion/Extension Bland-Altman Plots & Angle Values - Time Plot

Bland-Altman Plots

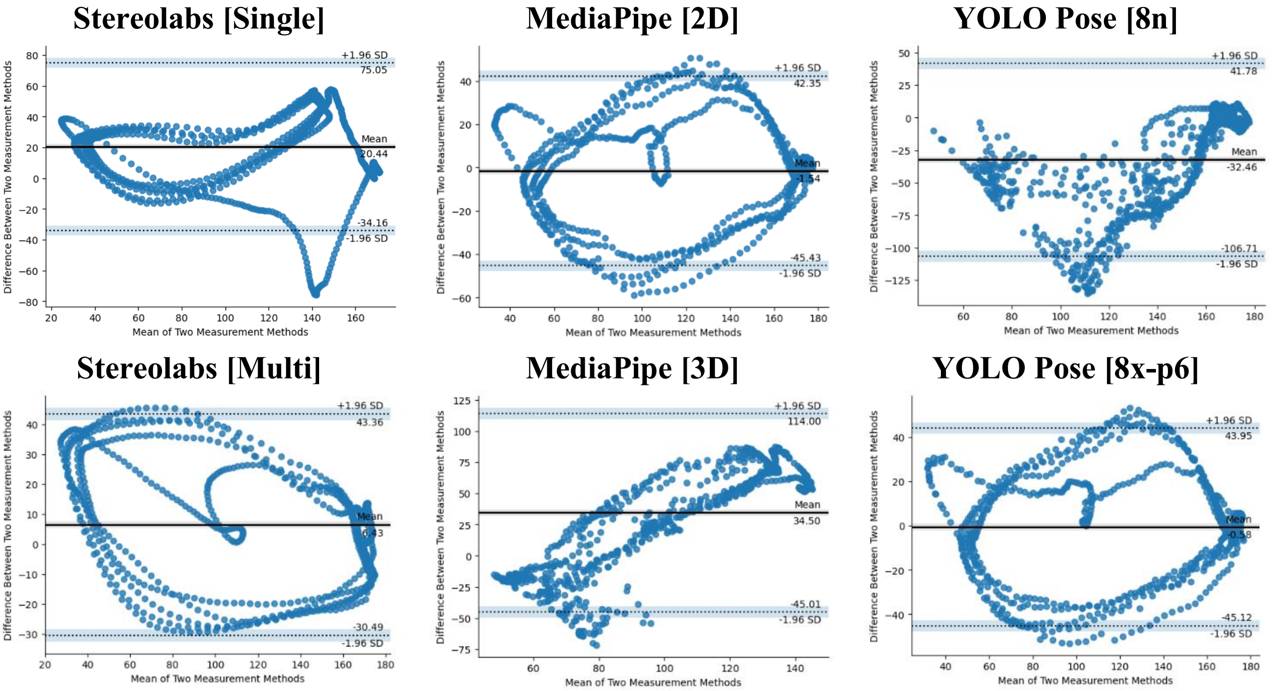

Angle Values - Time Plot

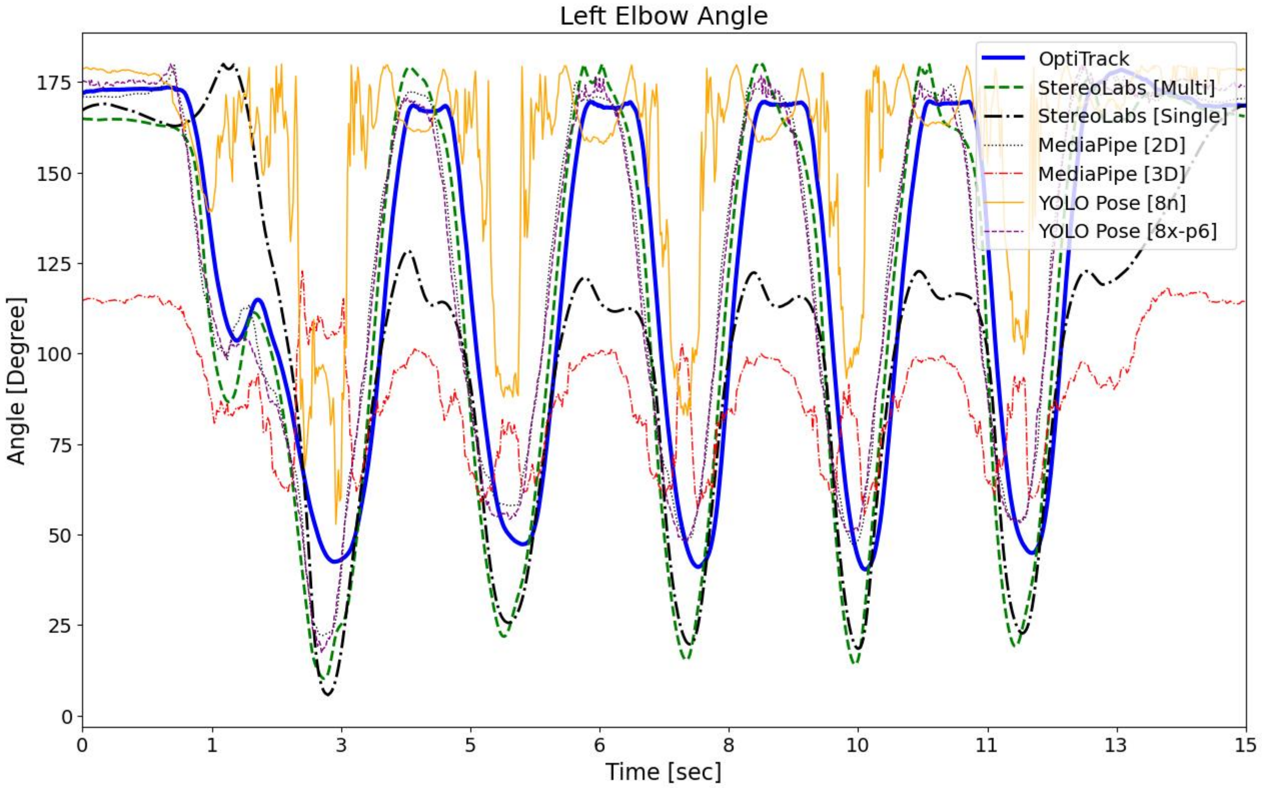

Supplementary Figure 8. Left Elbow Angle Flexion/Extension Bland-Altman Plots & Angle Values - Time Plot

Bland-Altman Plots

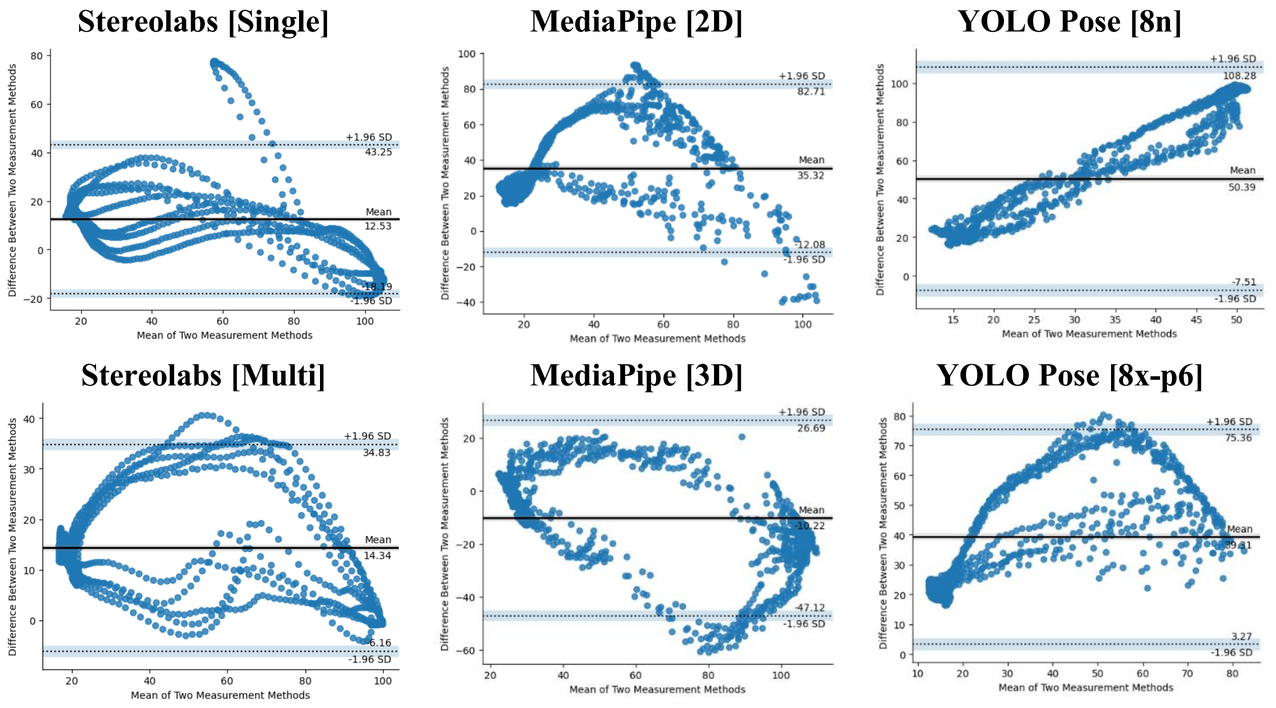

Angle Values - Time Plot

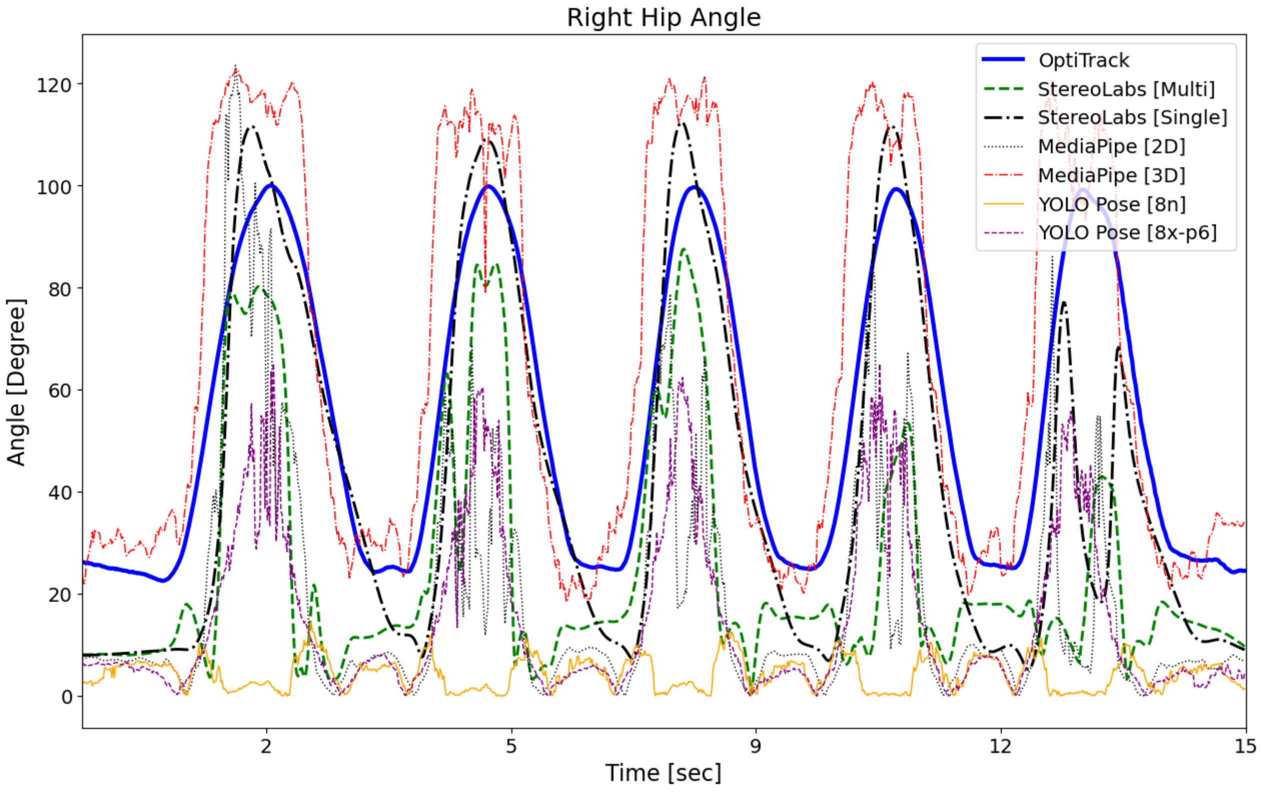

Supplementary Figure 9. Right Hip Angle Flexion/Extension Bland-Altman Plots & Angle Values - Time Plot

Bland-Altman Plots

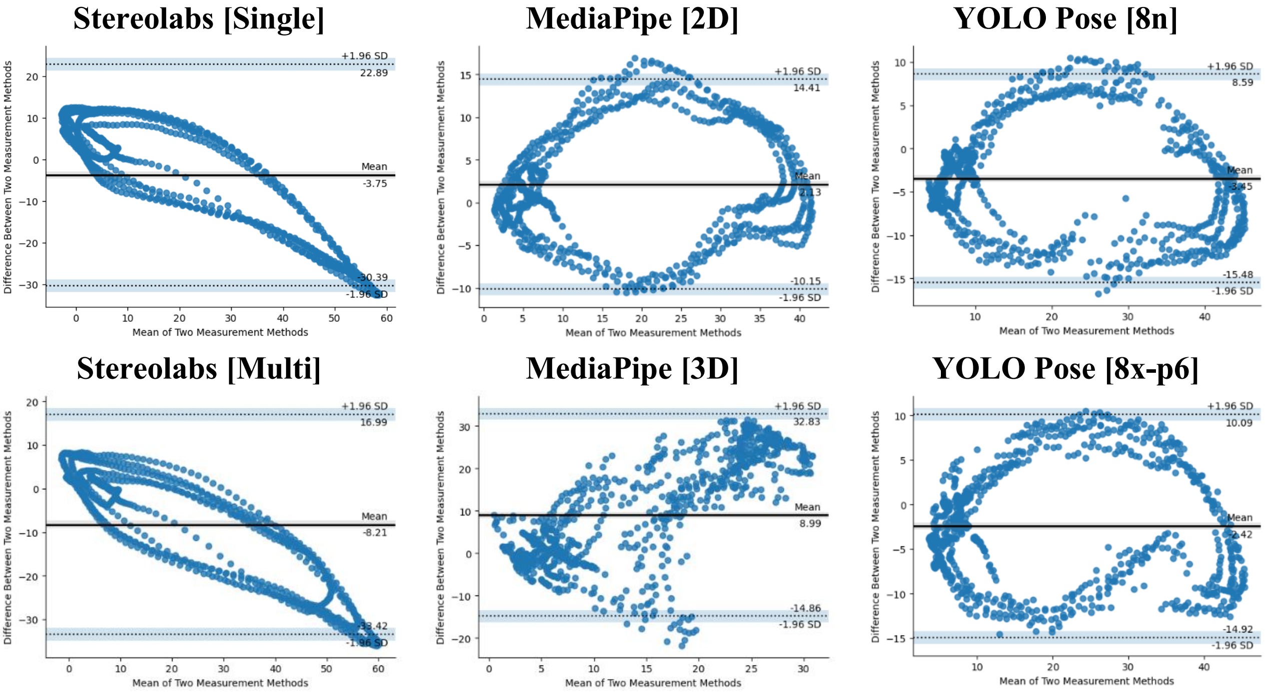

Angle Values - Time Plot

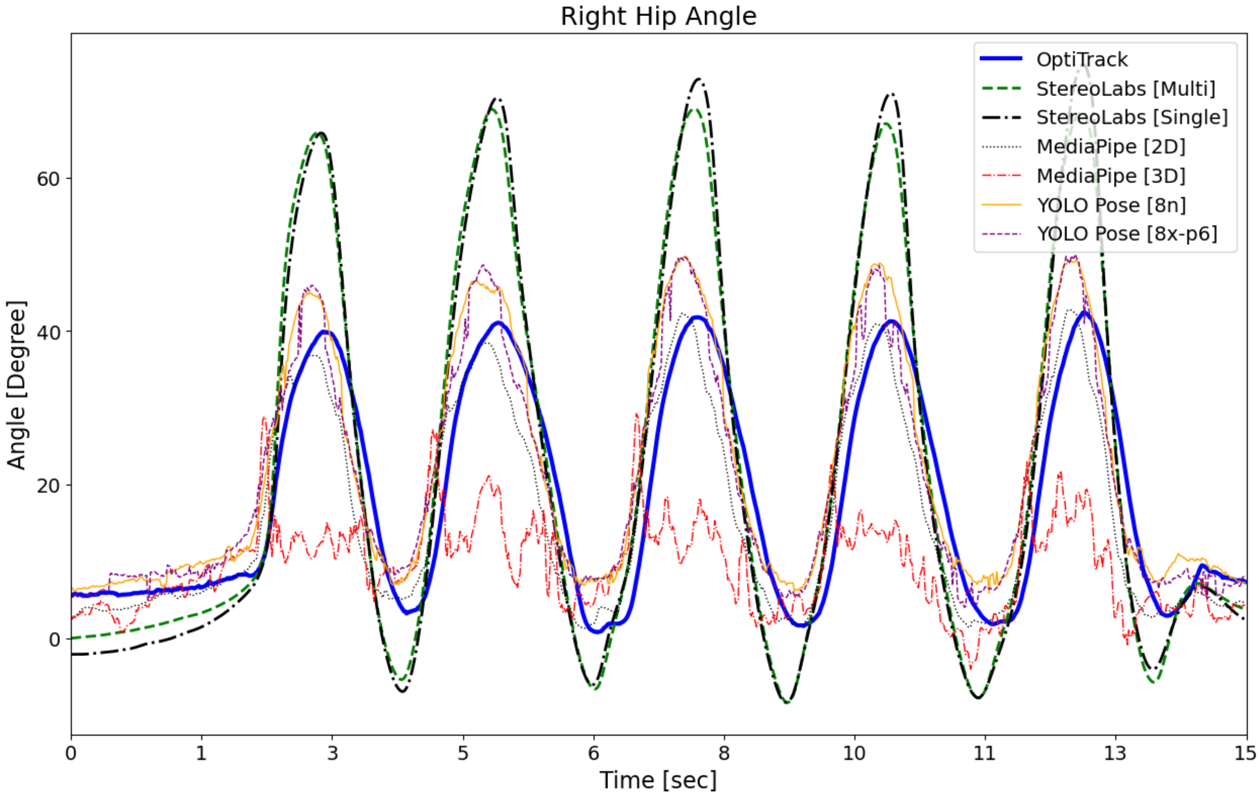

Supplementary Figure 10. Right Hip Angle Abduction/Adduction Bland-Altman Plots & Angle Values - Time Plot

Bland-Altman Plots

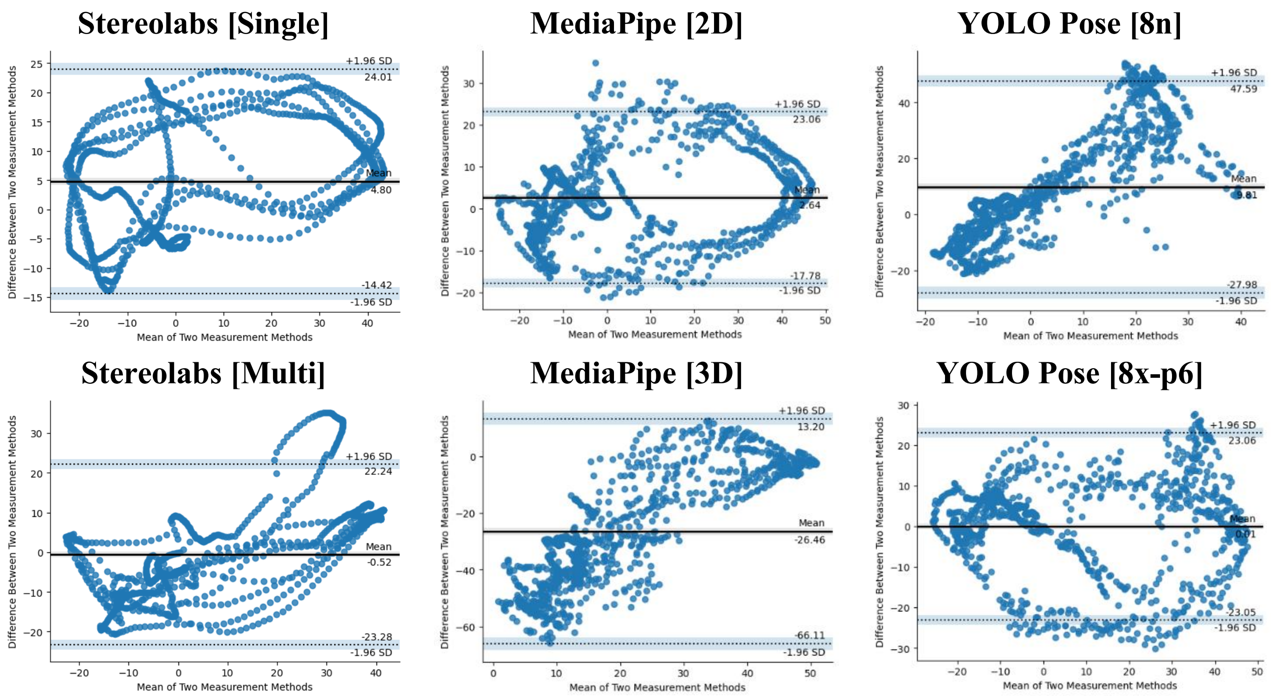

Angle Values - Time Plot

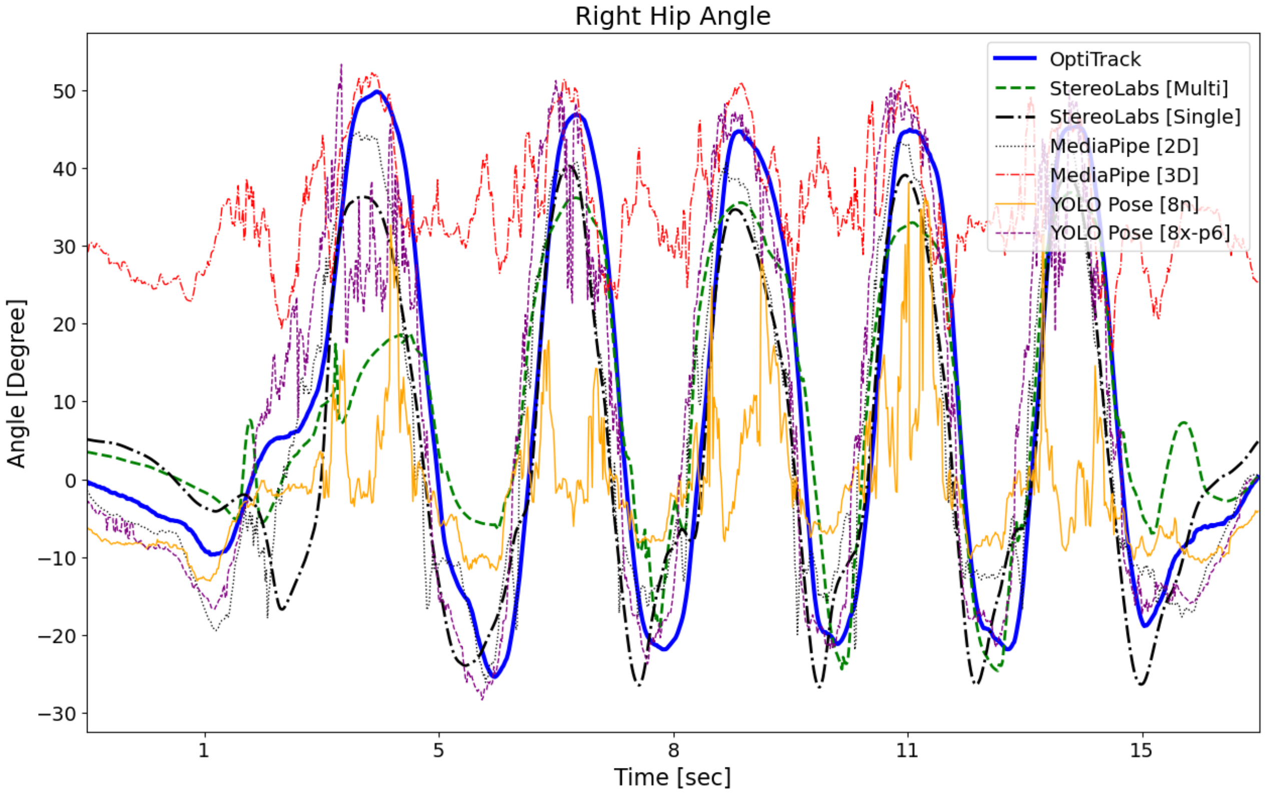

Supplementary Figure 11. Right Hip Angle Rotation Bland-Altman Plots & Angle Values - Time Plot

Bland-Altman Plots

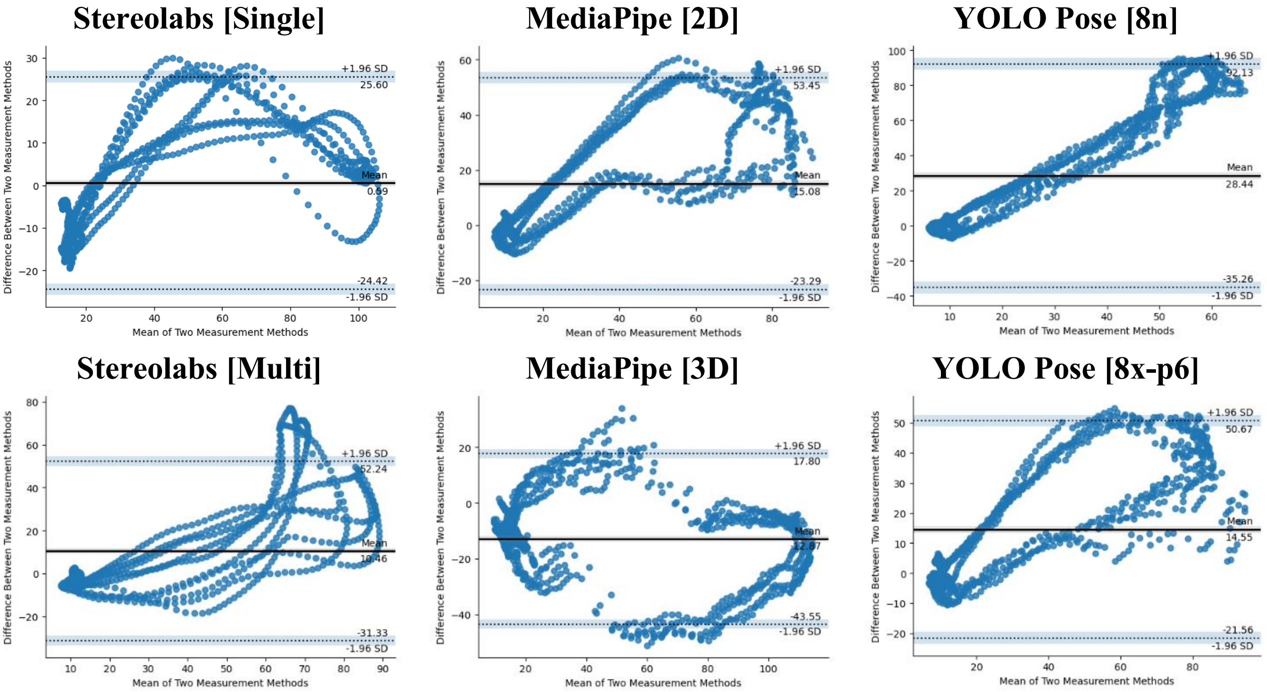

Angle Values - Time Plot

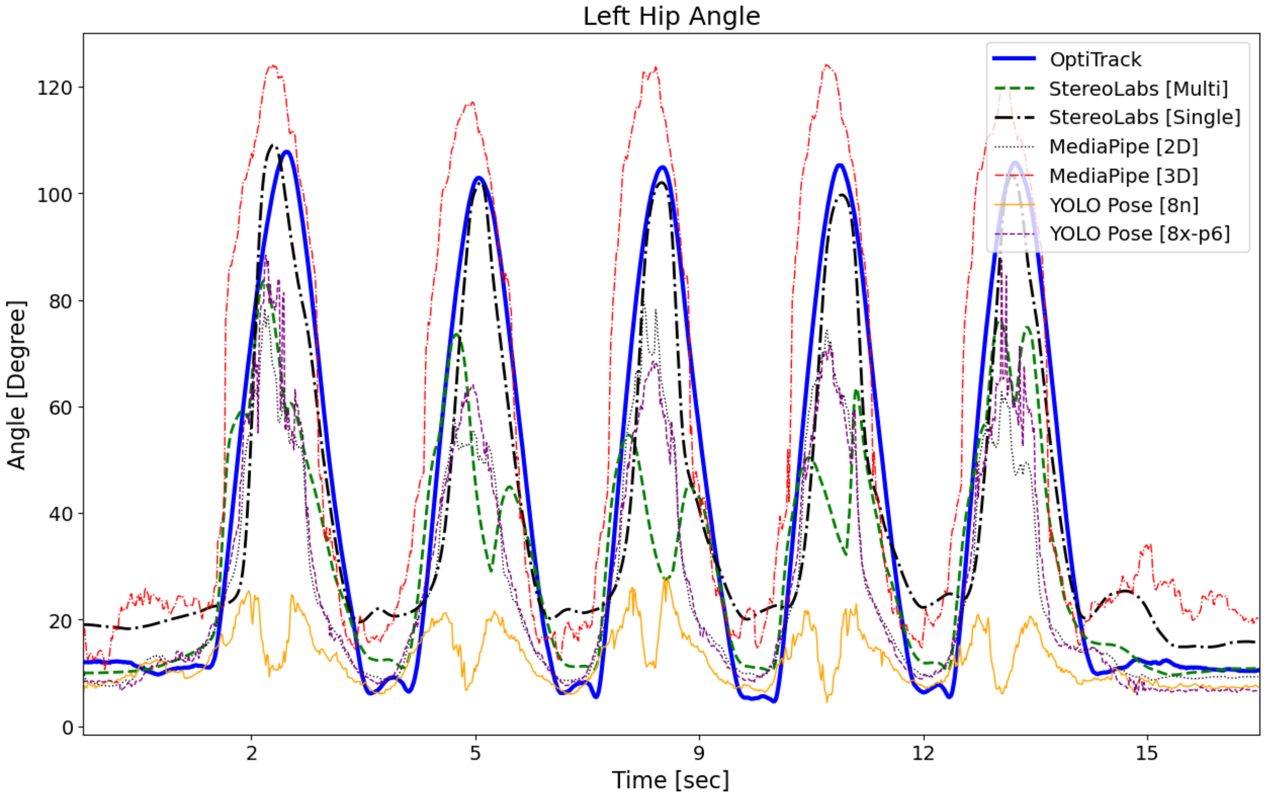

Supplementary Figure 12. Left Hip Angle Flexion/Extension Bland-Altman Plots & Angle Values - Time Plot

Bland-Altman Plots

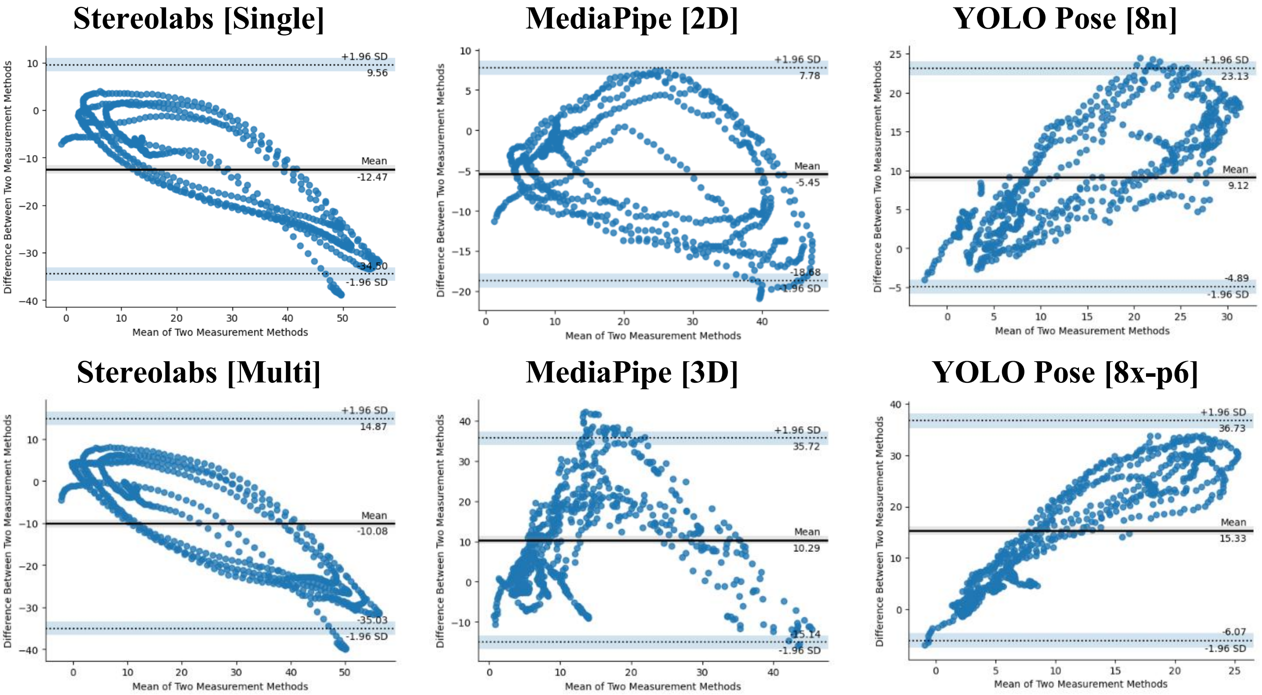

Angle Values - Time Plot

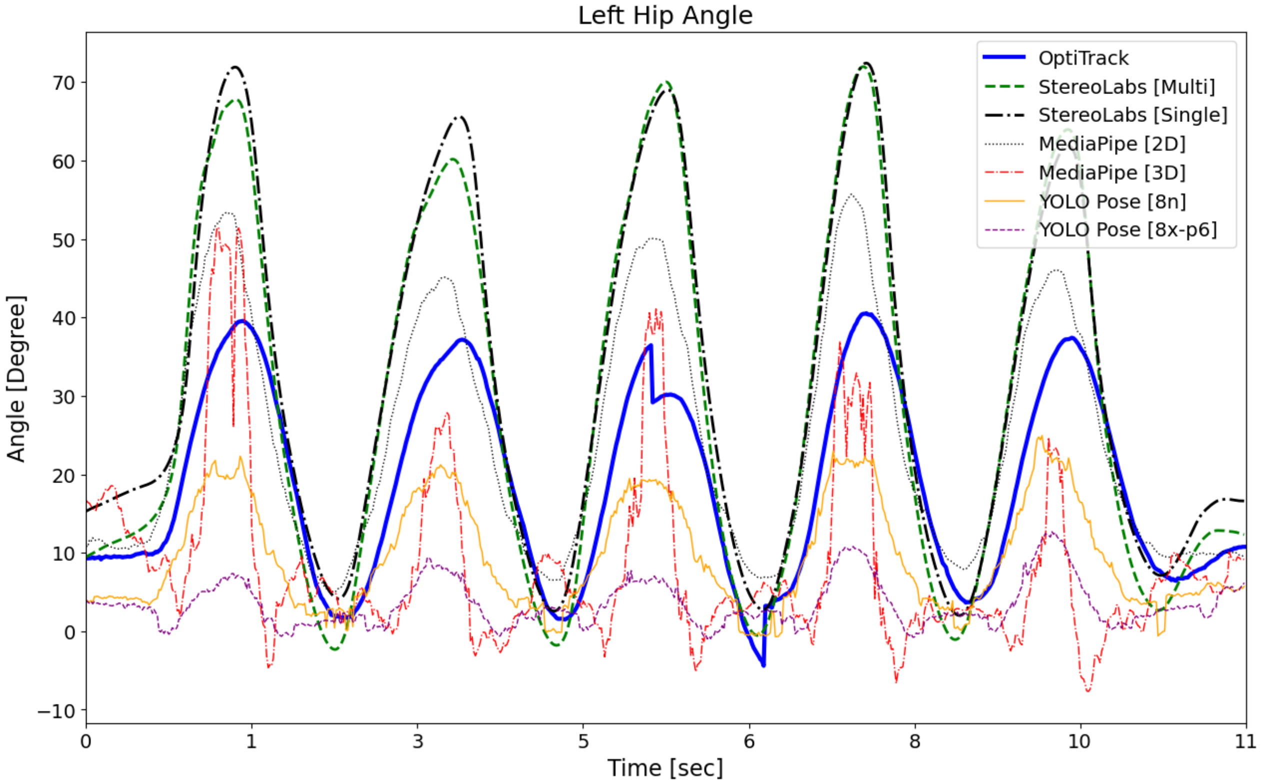

Supplementary Figure 13. Left Hip Angle Abduction/Adduction Bland-Altman Plots & Angle Values - Time Plot

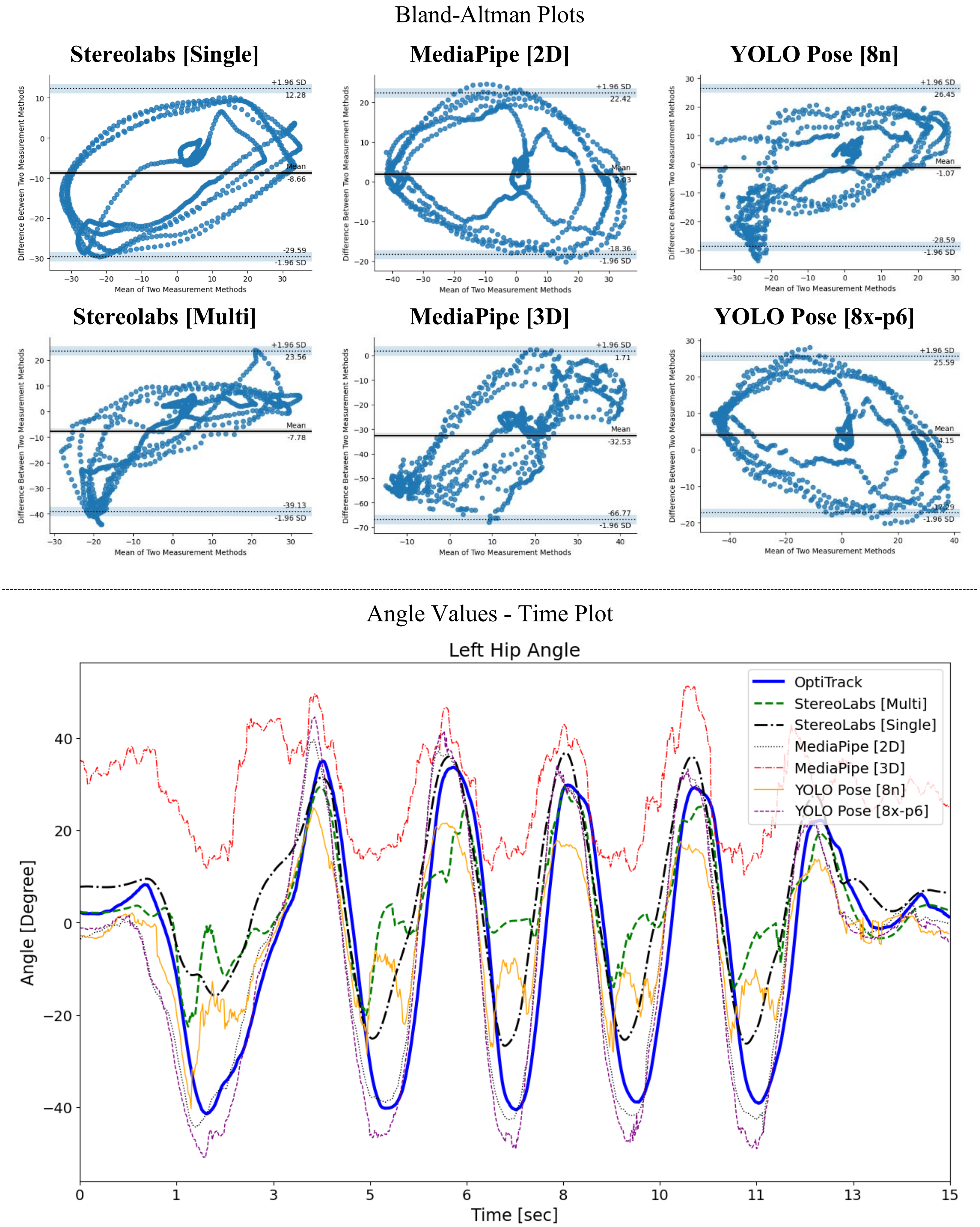

Supplementary Figure 14. Left Hip Angle Rotation Bland-Altman Plots & Angle Values - Time Plot

Bland-Altman Plots

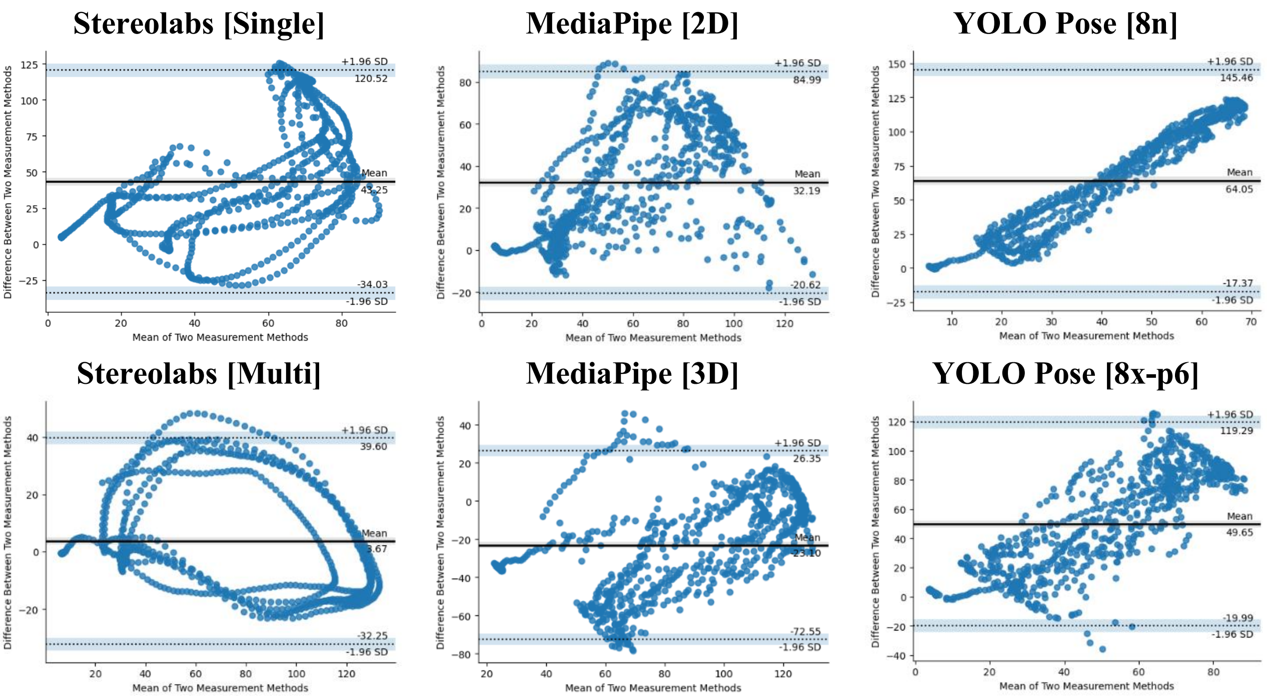

Angle Values - Time Plot

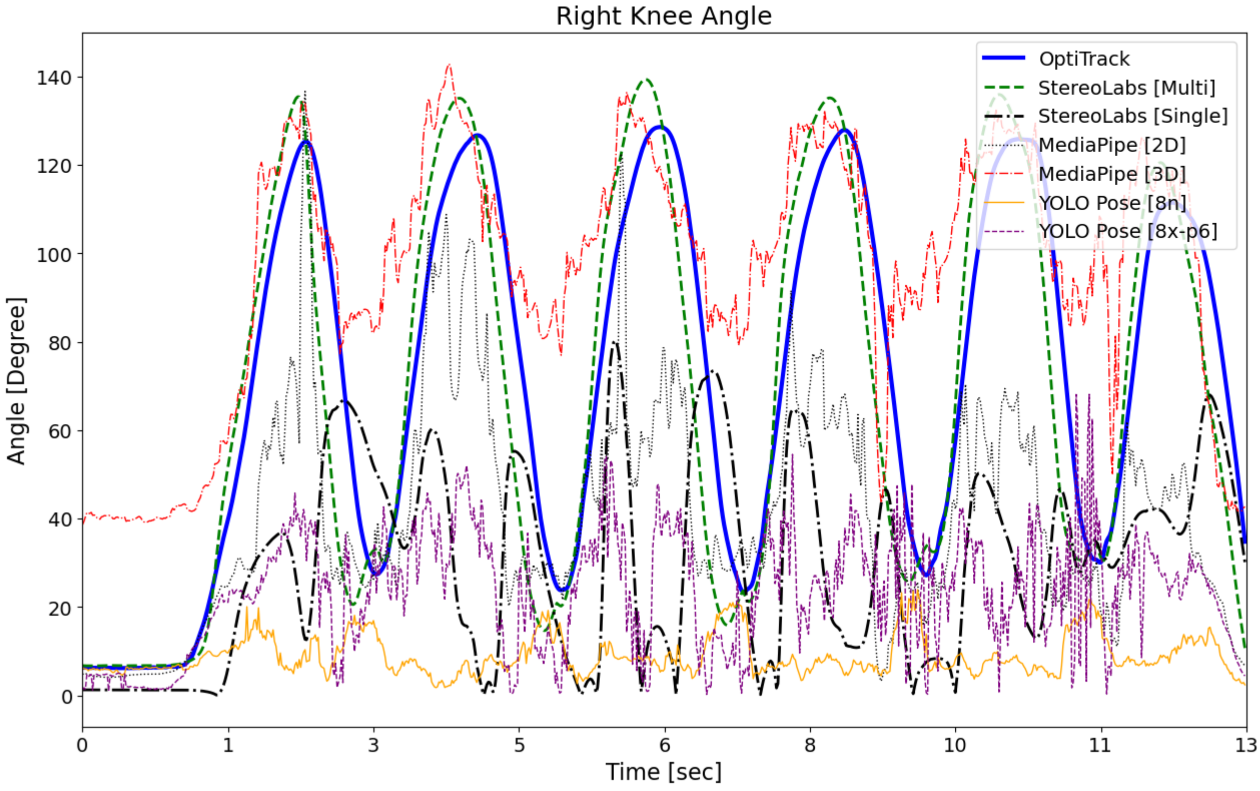

Supplementary Figure 15. Right Knee Angle Flexion/Extension Bland-Altman Plots & Angle Values - Time Plot

Bland-Altman Plots

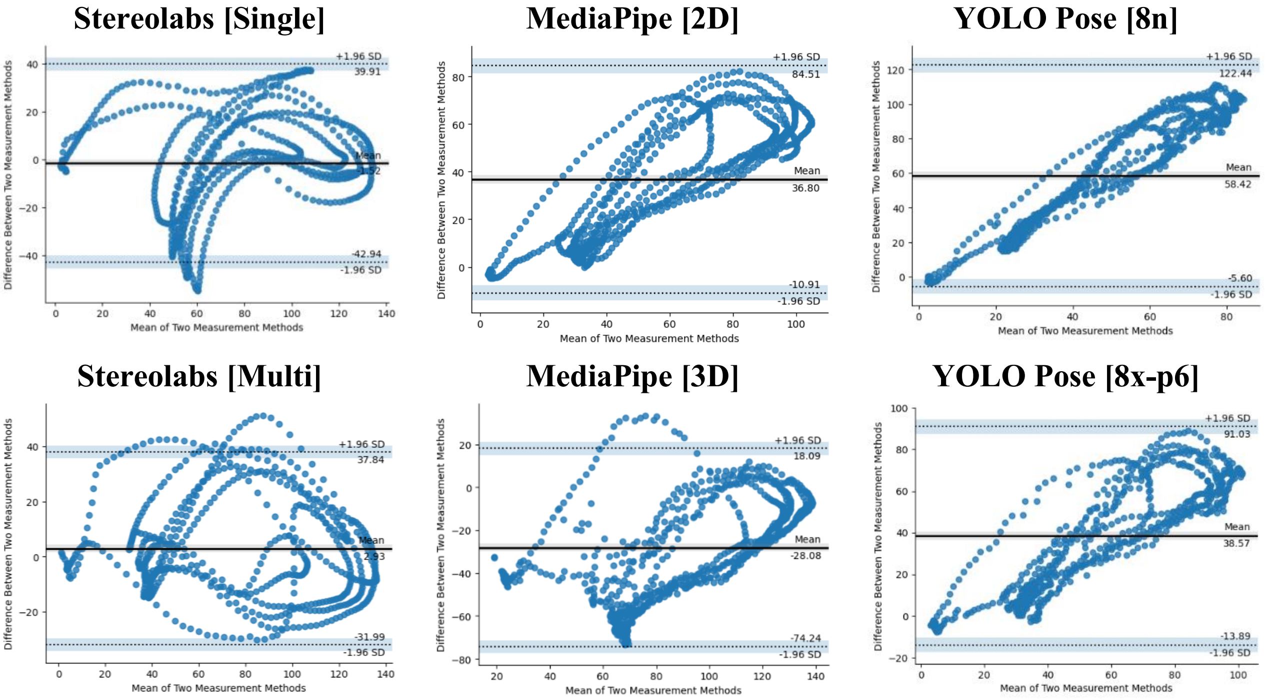

Angle Values - Time Plot

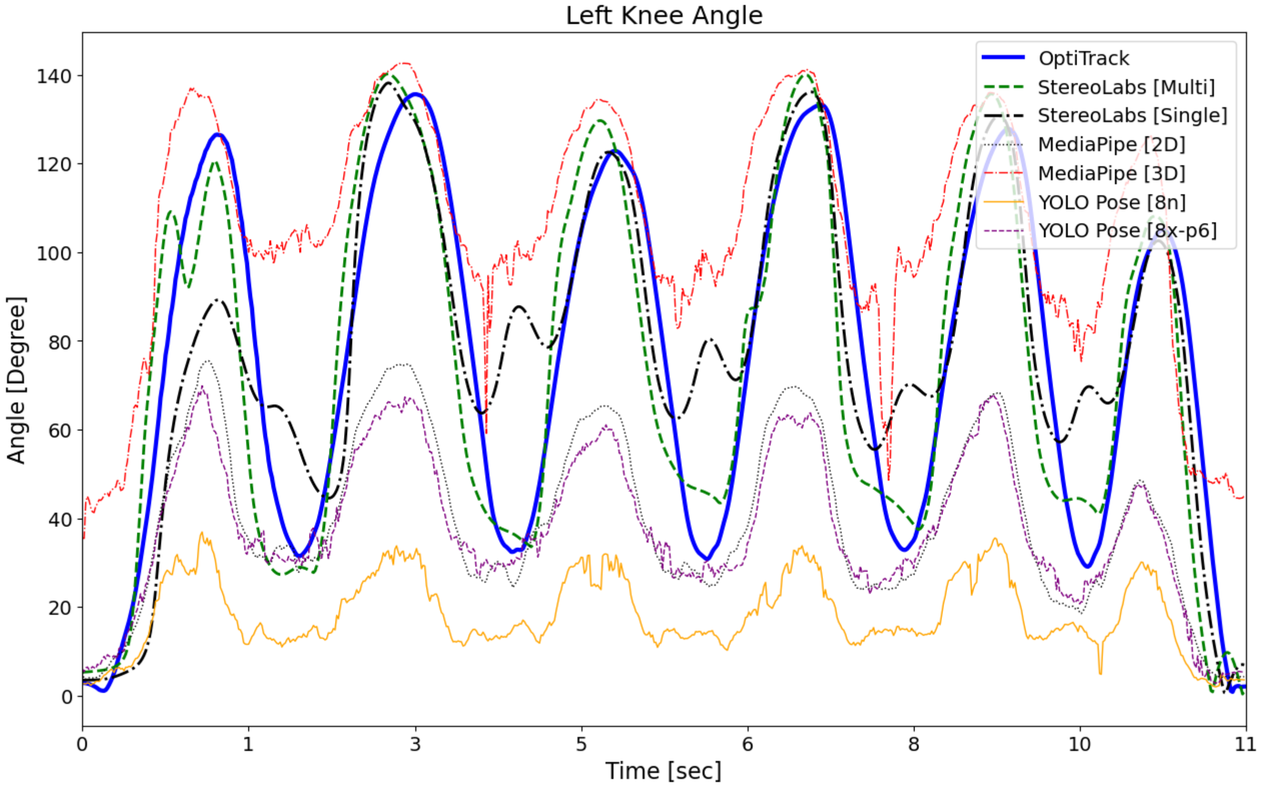

Supplementary Figure 16. Left Knee Angle Flexion/Extension Bland-Altman Plots & Angle Values - Time Plot

Supplementary Table 1. Reliability between methods w.r.t. golden standard for shoulder

Motion	Metric	StereoLabs [Multi]	StereoLabs [Single]	MediaPipe [2D]	MediaPipe [3D]	YOLO Pose [8n]	YOLO Pose [8x-p6]
**RSAA**	**Bias**	-2.03	6.55	3.82	7.27	5.22	3.87
	**LoA**	-32.23 to 28.17	-16.86 to 29.96	-24.24 to 31.88	-31.27 to 45.81	-22.97 to 33.42	-23.77 to 31.51
	**LoA Ratio**	0.99	0.97	0.98	0.92	0.98	0.98
	**ICC**	0.98	0.98	0.98	0.96	0.98	0.98
	**95% CI**	0.98 to 0.98	0.96 to 0.99	0.98 to 0.98	0.95 to 0.97	0.97 to 0.98	0.98 to 0.98
	**SEM**	2.18	1.56	1.97	3.73	2.18	1.99
	**MDC**	6.04	4.32	5.47	10.34	6.05	5.53
**RSHAA**	**Bias**	-11.19	-13.18	-28.71	-1.15	-22.23	-21.18
	**LoA**	-40.15 to 17.77	-58.97 to 32.61	-91.89 to 34.47	-71.71 to 69.41	-76.90 to 32.45	-90.39 to 48.04
	**LoA Ratio**	0.97	0.91	0.96	0.99	0.96	0.96
	**ICC**	0.94	0.88	0.77	0.32	0.82	0.79
	**95% CI**	0.78 to 0.97	0.76 to 0.93	0.28 to 0.89	0.25 to 0.39	0.46 to 0.91	0.59 to 0.87
	**SEM**	3.74	8.09	15.43	29.69	11.87	16.18
	**MDC**	10.36	22.43	42.76	82.29	32.90	44.86
**RSR**	**Bias**	-4.89	21.44	-0.81	-23.45	3.80	-3.79
	**LoA**	-37.13 to 27.34	-25.81 to 68.70	-68.33 to 66.72	-77.79 to 30.90	-96.42 to 104.02	-119.40 to 111.83
	**LoA Ratio**	0.93	0.98	0.9	1	1	1
	**ICC**	0.98	0.91	0.94	0.89	0.61	0.42
	**95% CI**	0.98 to 0.99	0.62 to 0.96	0.93 to 0.95	0.58 to 0.95	0.56 to 0.65	0.35 to 0.48
	**SEM**	2.21	7.07	8.30	9.24	31.97	44.96
	**MDC**	6.117	19.60	23.00	25.60	88.62	124.63
**LSAA**	**Bias**	-1.35	1.69	0.67	9.92	3.61	2.24
	**LoA**	-36.72 to 34.03	-37.40 to 40.78	-44.71 to 46.04	-42.44 to 62.27	-40.02 to 47.24	-43.15 to 47.62
	**LoA Ratio**	1	0.98	0.98	0.97	0.98	0.99
	**ICC**	0.97	0.96	0.95	0.93	0.95	0.95
	**95% CI**	0.97 to 0.97	0.96 to 0.97	0.94 to 0.96	0.90 to 0.95	0.94 to 0.96	0.94 to 0.95
	**SEM**	3.02	3.89	5.18	7.17	5.03	5.23
	**MDC**	8.37	10.78	14.35	19.87	13.93	14.49
**LSHAA**	**Bias**	-5.61	-1.08	4.95	6.63	4.00	-1.75
	**LoA**	-34.24 to 23.02	-27.28 to 25.11	-43.38 to 53.28	-39.95 to 53.21	-40.85 to 48.85	-62.21 to 58.71
	**LoA Ratio**	0.96	0.93	0.91	0.95	0.92	0.93
	**ICC**	0.96	0.97	0.88	0.82	0.86	0.85
	**95% CI**	0.94 to 0.97	0.96 to 0.97	0.86 to 0.89	0.78 to 0.85	0.84 to 0.87	0.84 to 0.87
	**SEM**	2.85	2.39	8.61	10.00	8.71	11.83
	**MDC**	7.89	6.63	23.87	27.72	24.15	32.78
**LSR**	**Bias**	-5.63	-13.04	-0.57	-20.94	-25.09	-1.23
	**LoA**	-40.23 to 28.98	-49.17 to 23.10	-49.47 to 48.33	-84.94 to 43.07	-94.19 to 44.01	-54.75 to 52.28
	**LoA Ratio**	0.92	0.99	0.95	1	0.98	0.94
	**ICC**	0.98	0.96	0.96	0.82	0.84	0.95
	**95% CI**	0.97 to 0.98	0.87 to 0.98	0.95 to 0.96	0.60 to 0.90	0.57 to 0.92	0.95 to 0.96
	**SEM**	2.68	3.69	5.05	14.01	14.32	6.04
	**MDC**	7.42	10.22	14.00	33.62	33.28	16.58

RSAA: Right Shoulder Abduction/Adduction, RSHAA: Right Shoulder Horizontal Abduction/Adduction, RSR: Right Shoulder Rotation, LSAA: Left Shoulder Abduction/Adduction, LSHAA: Left Shoulder Horizontal Abduction/Adduction, LSR: Left Shoulder Rotation

Supplementary Table 2. Reliability between methods w.r.t. golden standard for elbow

Motion	Metric	StereoLabs [Multi]	StereoLabs [Single]	MediaPipe [2D]	MediaPipe [3D]	YOLO Pose [8n]	YOLO Pose [8x-p6]
**REFE**	**Bias**	-3.47	-0.92	-3.87	10.13	-14.386	-3.74
	**LoA**	-34.52 to 27.59	-16.87 to 14.89	-44.24 to 36.51	-61.89 to 82.16	-59.85 to 31.07	-44.42 to 36.94
	**LoA Ratio**	0.9	0.99	0.94	1	0.95	0.94
	**ICC**	0.97	0.99	0.95	0.61	0.89	0.95
	**95% CI**	0.97 to 0.98	0.99 to 0.99	0.94 to 0.95	0.54 to 0.67	0.75 to 0.94	0.94 to 0.95
	**SEM**	2.70	0.68	4.79	22.92	7.69	4.87
	**MDC**	7.48	1.88	13.27	63.53	21.32	13.49
**LEFE**	**Bias**	6.44	20.45	-1.54	34.50	-32.47	-0.58
	**LoA**	-30.49 to 43.36	-34.16 to 75.05	-45.43 to 42.35	-45.01 to 114.00	-106.71 to 41.78	-45.12 to 43.95
	**LoA Ratio**	0.99	0.96	0.95	0.95	0.94	0.94
	**ICC**	0.96	0.86	0.94	0.42	0.57	0.94
	**95% CI**	0.95 to 0.97	0.61 to 0.93	0.93 to 0.95	0.02 to 0.63	0.09 to 0.76	0.93 to 0.95
	**SEM**	3.62	10.31	5.35	30.90	24.75	5.47
	**MDC**	10.05	28.58	14.82	85.63	68.61	15.17

REFE: Right Elbow Flexion/Extension, LEFE: Left Elbow Flexion/Extension

Supplementary Table 3. Reliability between methods w.r.t. golden standard for hip

Motion	Metric	StereoLabs [Multi]	StereoLabs [Single]	MediaPipe [2D]	MediaPipe [3D]	YOLO Pose [8n]	YOLO Pose [8x-p6]
RHFE	Bias	14.34	12.53	35.32	-10.22	50.39	39.31
	LoA	-6.16 to 34.83	-18.192 to 43.25	-12.08 to 82.71	-47.12 to 26.69	-7.51 to 108.29	3.27 to 75.36
	LoA Ratio	0.96	0.95	0.95	0.94	1	0.99
	ICC	0.91	0.90	0.47	0.89	-0.03	0.44
	95% CI	0.14 to 0.97	0.64 to 0.95	-0.21 to 0.75	0.79 to 0.94	-0.10 to 0.04	-0.17 to 0.77
	SEM	3.10	5.01	17.64	6.16	29.99	13.73
	MDC	8.60	13.88	48.89	17.07	83.14	38.05
RHAA	Bias	-8.21	-3.75	2.13	8.99	-3.45	-2.42
	LoA	-33.42 to 16.99	-30.39 to 22.89	-10.15 to 14.41	-14.86 to 32.83	-15.48 to 8.59	-14.92 to 10.09
	LoA Ratio	0.97	0.97	0.97	0.98	0.97	0.99
	ICC	0.86	0.88	0.94	0.43	0.94	0.94
	95% CI	0.67 to 0.92	0.85 to 0.90	0.92 to 0.95	0.10 to 0.62	0.87 to 0.97	0.91 to 0.96
	SEM	4.88	4.79	1.55	9.19	1.50	1.56
	MDC	13.53	13.27	4.29	25.46	4.17	4.33
RHR	Bias	-0.52	4.80	2.64	-26.46	9.81	0.01
	LoA	-23.29 to 22.24	-14.42 to 24.01	-17.78 to 23.06	-66.11 to 13.20	-27.98 to 47.59	-23.05 to 23.06
	LoA Ratio	0.94	1	0.95	1	0.94	0.92
	ICC	0.91	0.93	0.94	0.27	0.52	0.93
	95% CI	0.90 to 0.92	0.88 to 0.96	0.92 to 0.95	-0.15 to 0.53	0.33 to 0.64	0.92 to 0.94
	SEM	3.50	2.52	2.59	17.35	13.40	3.02
	MDC	9.71	6.98	7.19	48.08	37.14	8.38
LHFE	Bias	10.46	0.59	15.08	-12.87	28.44	14.56
	LoA	-31.33 to 52.24	-24.42 to 25.60	-23.29 to 53.45	-43.55 to 17.80	-35.26 to 92.13	-21.56 to 50.67
	LoA Ratio	0.93	0.97	0.97	0.93	0.97	0.97
	ICC	0.81	0.96	0.81	0.93	0.20	0.83
	95% CI	0.69 to 0.87	0.95 to 0.96	0.46 to 0.90	0.71 to 0.97	-0.03 to 0.37	0.49 to 0.92
	SEM	9.24	2.62	8.62	4.26	29.14	7.55
	MDC	25.62	7.25	23.90	11.80	80.77	20.94
LHAA	Bias	-10.08	-12.47	-5.450	10.29	9.12	15.33
	LoA	-35.03 to 14.87	-34.50 to 9.56	-18.68 to 7.78	-15.14 to 35.72	-4.89 to 23.13	-6.07 to 36.73
	LoA Ratio	0.98	0.97	0.98	0.95	0.98	1
	ICC	0.80	0.79	0.9	0.46	0.69	0.2
	95% CI	0.43 to 0.90	0.07 to 0.92	0.64 to 0.96	0.07 to 0.66	-0.13 to 0.88	-0.13 to 0.45
	SEM	5.65	5.21	2.13	9.55	3.95	9.77
	MDC	15.66	14.45	5.92	26.48	10.96	27.07
LHR	Bias	-7.78	-8.66	2.03	-32.53	-1.07	4.15
	LoA	-39.13 to 23.56	-29.59 to 12.28	-18.36 to 22.42	-66.77 to 1.71	-28.59 to 26.45	-17.29 to 25.59
	LoA Ratio	0.95	1	0.98	0.99	0.96	0.98
	ICC	0.71	0.88	0.95	0.32	0.84	0.94
	95% CI	0.56 to 0.80	0.59 to 0.95	0.94 to 0.95	-0.18 to 0.65	0.81 to 0.86	0.91 to 0.96
	SEM	8.60	3.67	2.40	14.37	5.70	2.66
	MDC	23.83	10.17	6.64	39.84	15.81	7.37

RHFE: Right Hip Flexion/Extension, RHAA: Right Hip Abduction/Adduction, RHR: Right Hip Rotation, LHFE: Left Hip Flexion/Extension, LHAA: Left Hip Abduction/Adduction, LHR: Left Hip Rotation

Supplementary Table 4. Reliability between methods w.r.t. golden standard for knee

Motion	Metric	StereoLabs [Multi]	StereoLabs [Single]	MediaPipe [2D]	MediaPipe [3D]	YOLO Pose [8n]	YOLO Pose [8x-p6]
RKFE	Bias	3.68	43.25	32.19	-23.10	64.05	49.65
	LoA	-32.26 to 39.60	-34.03 to 120.52	-20.62 to 84.99	-72.55 to 26.35	-17.37 to 145.46	-19.99 to 119.29
	LoA Ratio	0.98	0.98	0.99	0.96	1	0.99
	ICC	0.95	0.24	0.63	0.76	-0.03	0.22
	95% CI	0.94 to 0.96	-0.10 to 0.47	-0.12 to 0.84	0.22 to 0.89	-0.10 to 0.04	-0.14 to 0.48
	SEM	4.14	34.32	16.30	12.49	42.18	31.30
	MDC	11.48	95.14	45.18	34.62	116.91	86.76
LKFE	Bias	2.93	-1.52	36.80	-28.08	58.42	38.57
	LoA	-31.99 to 37.84	-42.94 to 39.91	-10.91 to 84.51	-74.24 to 18.09	-5.60 to 122.44	-13.89 to 91.03
	LoA Ratio	0.95	0.96	1	0.98	1	1
	ICC	0.95	0.91	0.57	0.71	0.18	0.49
	95% CI	0.94 to 0.95	0.89 to 0.92	-0.22 to 0.82	-0.08 to 0.89	-0.14 to 0.45	-0.21 to 0.77
	SEM	4.18	6.38	16.00	12.66	29.51	19.12
	MDC	11.58	17.67	44.35	35.10	81.79	52.98

RKFE: Right Knee Flexion/Extension, LKFE: Left Knee Flexion/Extension
